# Supplementary figures and images for: Integrating economic measures of adaptation effectiveness into climate change interventions: A case study of irrigation development in Mwea, Kenya
Source: PLoS One. 2020 Dec 11;15(12):e0243779. doi: 10.1371/journal.pone.0243779 (PMC7732349; doi:10.1371/journal.pone.0243779)

**S1 File.** **Estimated crop coefficients**

**
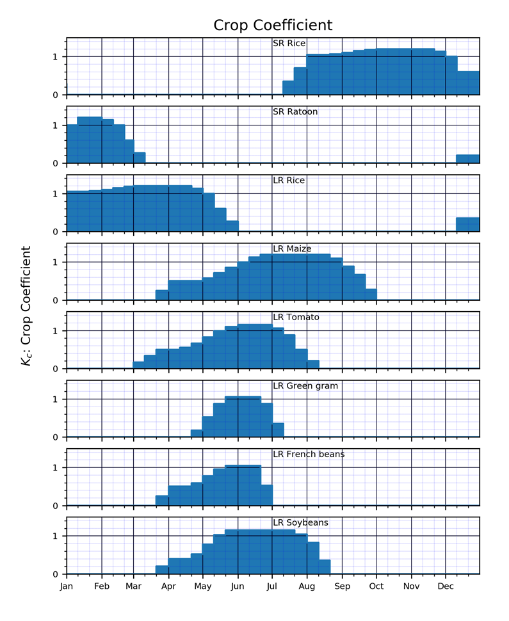
**

Supplement: S1 File — (DOCX) [file pone.0243779.s001.docx]
