# Supplementary material for: Integrating economic measures of adaptation effectiveness into climate change interventions: A case study of irrigation development in Mwea, Kenya
Source: PLoS One. 2020 Dec 11;15(12):e0243779. doi: 10.1371/journal.pone.0243779 (PMC7732349; doi:10.1371/journal.pone.0243779)
Supplement: S2 File — (DOCX) [file pone.0243779.s002.docx]

**S2 File.** **Yield functions**

Crop yields are first estimated by the DSSAT model in the conditions of Mwea for a set of combinations of temperature change and irrigation water sufficiency (7 x 11 = 77 combinations). By multivariate regression, the obtained values from the model are approximated in the following polynomial function of the percentage yield change from the baseline (i.e., the baseline/current yield with the respective water sufficiency rate), where *x* and *y* are the change in annual temperature from the present (˚C) and the irrigation water sufficiency rate (%), respectively:

$$f\left( x,y \right)=a_{1}+a_{2}x+a_{3}y+a_{4}x^{2}+a_{5}x^{2}y+a_{6}x^{2}y^{2}+a_{7}y^{2}+a_{8}{xy}^{2}+a_{9}xy+a_{10}x^{3}+a_{11}y^{3}$$

A table of coefficients by crop is presented below. Note that coefficients are different for different RCP scenarios because the estimates reflect the carbon dioxide fertilization effect.

SR: Short Rainy Season; LR: Long Rainy Season

| Target Year | RCP | Crop | Parameter Estimates | | | | | | | | | | |
| --- | --- | --- | --- | --- | --- | --- | --- | --- | --- | --- | --- | --- | --- |
|  |  |  | a_1_ | a_2_ | a_3_ | a_4_ | a_5_ | a_6_ | a_7_ | a_8_ | a_9_ | a_10_ | a_11_ |
| 2030 | rcp26 | SR Rice | 1.16E+01 | -3.14E+01 | -2.97E-01 | 1.75E+00 | -5.97E-02 | 2.29E-04 | 4.88E-03 | -1.01E-03 | 3.77E-01 | 4.17E-01 | -2.74E-05 |
|  |  | LR Rice | 5.22E+00 | -3.07E+01 | 1.23E-01 | 5.09E+00 | -5.03E-02 | 2.29E-04 | -2.21E-03 | -6.43E-04 | 2.78E-01 | -4.13E-01 | 8.19E-06 |
|  |  | LR French beans | 1.04E+01 | -1.22E+00 | -3.50E-02 | -3.78E-01 | 4.47E-02 | -4.28E-04 | 9.19E-04 | 1.32E-03 | -1.28E-01 | -2.40E-01 | -9.36E-06 |
|  |  | LR Maize | 3.04E+00 | 7.76E+00 | -2.67E-01 | 1.58E+00 | 3.56E-02 | -3.36E-04 | 7.43E-03 | 2.94E-03 | -4.01E-01 | -6.17E-01 | -5.10E-05 |
|  |  | LR Soybeans | 1.14E+01 | 1.00E+00 | -8.45E-02 | -3.30E-01 | 4.97E-03 | 9.97E-06 | 2.69E-03 | 4.10E-04 | -5.16E-02 | 7.25E-02 | -1.91E-05 |
|  |  | LR Tomato | 1.11E+01 | -8.02E+00 | -1.83E-01 | -4.01E-02 | -1.43E-02 | 1.55E-04 | 1.15E-03 | -1.14E-03 | 1.38E-01 | -1.71E-01 | 3.15E-06 |
|  | rcp45 | SR Rice | 1.34E+01 | -3.24E+01 | -3.06E-01 | 2.04E+00 | -5.96E-02 | 2.07E-04 | 4.36E-03 | -1.05E-03 | 3.89E-01 | 3.79E-01 | -2.23E-05 |
|  |  | LR Rice | 5.63E+00 | -3.00E+01 | 9.22E-02 | 4.45E+00 | -5.38E-02 | 2.70E-04 | -1.46E-03 | -8.01E-04 | 2.91E-01 | -2.71E-01 | 3.66E-06 |
|  |  | LR French beans | 1.14E+01 | -8.12E-01 | -7.51E-03 | -6.83E-01 | 5.26E-02 | -5.03E-04 | 4.19E-04 | 1.60E-03 | -1.56E-01 | -1.83E-01 | -7.38E-06 |
|  |  | LR Maize | 3.38E+00 | 7.46E+00 | -2.90E-01 | 1.77E+00 | 2.95E-02 | -2.78E-04 | 7.65E-03 | 2.71E-03 | -3.77E-01 | -6.52E-01 | -5.12E-05 |
|  |  | LR Soybeans | 1.24E+01 | 1.30E+00 | -7.50E-02 | -4.74E-01 | 8.99E-03 | -1.71E-05 | 2.51E-03 | 4.17E-04 | -5.52E-02 | 7.37E-02 | -1.80E-05 |
|  |  | LR Tomato | 1.21E+01 | -7.75E+00 | -1.82E-01 | -2.52E-01 | -1.12E-02 | 1.32E-04 | 1.08E-03 | -1.07E-03 | 1.30E-01 | -1.41E-01 | 3.52E-06 |
|  | rcp60 | SR Rice | 1.19E+01 | -3.16E+01 | -3.11E-01 | 1.64E+00 | -6.83E-02 | 3.13E-04 | 5.06E-03 | -1.28E-03 | 4.06E-01 | 4.50E-01 | -2.80E-05 |
|  |  | LR Rice | 6.50E+00 | -3.13E+01 | 1.23E-01 | 4.89E+00 | -6.00E-02 | 3.08E-04 | -2.87E-03 | -9.23E-04 | 3.17E-01 | -3.26E-01 | 1.33E-05 |
|  |  | LR French beans | 1.07E+01 | -1.41E+00 | -5.44E-02 | -4.08E-01 | 4.15E-02 | -4.12E-04 | 1.53E-03 | 1.30E-03 | -1.21E-01 | -2.08E-01 | -1.39E-05 |
|  |  | LR Maize | 3.34E+00 | 7.30E+00 | -2.95E-01 | 1.90E+00 | 3.51E-02 | -3.30E-04 | 7.74E-03 | 2.80E-03 | -3.87E-01 | -6.89E-01 | -5.14E-05 |
|  |  | LR Soybeans | 1.13E+01 | 1.44E+00 | -6.95E-02 | -5.86E-01 | 1.08E-02 | -3.48E-05 | 2.33E-03 | 5.12E-04 | -6.52E-02 | 9.78E-02 | -1.67E-05 |
|  |  | LR Tomato | 1.06E+01 | -6.23E+00 | -1.49E-01 | -1.32E+00 | 6.70E-03 | -4.24E-05 | 5.62E-04 | -6.96E-04 | 8.94E-02 | 7.67E-02 | 5.96E-06 |
|  | rcp85 | SR Rice | 1.48E+01 | -3.47E+01 | -3.08E-01 | 3.33E+00 | -7.36E-02 | 2.69E-04 | 4.84E-03 | -1.03E-03 | 4.07E-01 | 2.53E-01 | -2.75E-05 |
|  |  | LR Rice | 7.36E+00 | -2.99E+01 | 1.20E-01 | 3.90E+00 | -5.59E-02 | 2.71E-04 | -2.24E-03 | -7.55E-04 | 2.96E-01 | -1.08E-01 | 7.79E-06 |
|  |  | LR French beans | 1.40E+01 | -1.33E+00 | -4.98E-02 | -3.38E-01 | 5.10E-02 | -4.98E-04 | 1.20E-03 | 1.55E-03 | -1.47E-01 | -2.46E-01 | -1.21E-05 |
|  |  | LR Maize | 3.86E+00 | 7.43E+00 | -3.04E-01 | 1.82E+00 | 2.97E-02 | -2.89E-04 | 7.95E-03 | 2.76E-03 | -3.79E-01 | -6.61E-01 | -5.32E-05 |
|  |  | LR Soybeans | 1.53E+01 | 1.01E+00 | -8.36E-02 | -4.01E-01 | 2.24E-03 | 3.83E-05 | 2.59E-03 | 2.45E-04 | -3.26E-02 | 7.84E-02 | -1.83E-05 |
|  |  | LR Tomato | 1.37E+01 | -6.22E+00 | -1.84E-01 | -1.47E+00 | 6.21E-03 | -6.70E-05 | 1.11E-03 | -5.54E-04 | 8.19E-02 | 1.56E-01 | 2.56E-06 |
| 2050 | rcp26 | SR Rice | 1.27E+01 | -3.41E+01 | -2.92E-01 | 3.10E+00 | -7.60E-02 | 3.30E-04 | 4.92E-03 | -1.28E-03 | 4.21E-01 | 2.59E-01 | -2.84E-05 |
|  |  | LR Rice | 6.43E+00 | -2.95E+01 | 5.10E-02 | 4.30E+00 | -4.12E-02 | 1.48E-04 | -4.26E-04 | -5.32E-04 | 2.64E-01 | -2.86E-01 | -3.15E-06 |
|  |  | LR French beans | 1.21E+01 | -9.69E-01 | -3.71E-02 | -3.30E-01 | 4.96E-02 | -4.43E-04 | 8.44E-04 | 1.38E-03 | -1.44E-01 | -2.83E-01 | -8.59E-06 |
|  |  | LR Maize | 3.50E+00 | 7.28E+00 | -2.94E-01 | 2.08E+00 | 3.68E-02 | -3.51E-04 | 7.94E-03 | 2.95E-03 | -4.03E-01 | -7.30E-01 | -5.36E-05 |
|  |  | LR Soybeans | 1.39E+01 | 8.90E-01 | -9.08E-02 | -3.55E-01 | 3.76E-03 | 2.85E-05 | 2.69E-03 | 2.75E-04 | -3.80E-02 | 7.00E-02 | -1.87E-05 |
|  |  | LR Tomato | 1.25E+01 | -6.76E+00 | -1.80E-01 | -7.71E-01 | -9.42E-03 | 1.26E-04 | 1.01E-03 | -9.21E-04 | 1.10E-01 | -5.90E-02 | 3.99E-06 |
|  | rcp45 | SR Rice | 2.33E+01 | -3.97E+01 | -4.61E-01 | 4.02E+00 | -1.32E-01 | 7.48E-04 | 6.23E-03 | -2.69E-03 | 6.21E-01 | 3.88E-01 | -3.15E-05 |
|  |  | LR Rice | 1.36E+01 | -3.08E+01 | -2.17E-02 | 3.72E+00 | -5.40E-02 | 2.50E-04 | 2.41E-04 | -6.90E-04 | 3.04E-01 | -7.93E-02 | -7.13E-06 |
|  |  | LR French beans | 1.90E+01 | -1.41E+00 | -2.55E-01 | -6.99E-01 | 1.18E-02 | -1.07E-04 | 2.76E-03 | -2.70E-04 | 4.02E-02 | -1.31E-01 | -9.38E-06 |
|  |  | LR Maize | 3.97E+00 | 8.03E+00 | -3.06E-01 | 2.12E+00 | 3.18E-02 | -2.87E-04 | 8.42E-03 | 2.83E-03 | -3.98E-01 | -7.85E-01 | -5.72E-05 |
|  |  | LR Soybeans | 2.08E+01 | 5.30E+00 | -4.02E-01 | -4.24E+00 | -6.09E-03 | 1.52E-04 | 8.29E-03 | -5.52E-04 | 3.59E-02 | 9.46E-01 | -4.37E-05 |
|  |  | LR Tomato | 1.84E+01 | -6.21E+00 | -2.10E-01 | -1.14E+00 | 4.36E-02 | -3.78E-04 | 8.74E-04 | 2.04E-04 | -1.08E-02 | -1.33E-02 | 6.66E-06 |
|  | rcp60 | SR Rice | 1.96E+01 | -3.61E+01 | -4.28E-01 | 2.93E+00 | -1.03E-01 | 5.42E-04 | 6.72E-03 | -1.99E-03 | 5.15E-01 | 4.51E-01 | -3.64E-05 |
|  |  | LR Rice | 1.25E+01 | -3.10E+01 | 4.97E-03 | 3.98E+00 | -5.25E-02 | 2.29E-04 | -1.57E-04 | -6.19E-04 | 2.96E-01 | -1.32E-01 | -5.09E-06 |
|  |  | LR French beans | 1.88E+01 | -1.64E+00 | -2.68E-01 | -1.45E+00 | 9.48E-03 | -1.28E-04 | 2.95E-03 | -1.91E-04 | 5.04E-02 | 1.21E-01 | -1.09E-05 |
|  |  | LR Maize | 3.77E+00 | 8.01E+00 | -3.04E-01 | 2.10E+00 | 3.11E-02 | -2.76E-04 | 8.41E-03 | 2.80E-03 | -3.96E-01 | -7.81E-01 | -5.72E-05 |
|  |  | LR Soybeans | 2.05E+01 | 1.85E+00 | -1.72E-01 | -1.59E+00 | 5.27E-02 | -5.22E-04 | 4.82E-03 | 1.93E-03 | -1.81E-01 | 3.93E-01 | -3.35E-05 |
|  |  | LR Tomato | 1.78E+01 | -6.89E+00 | -2.28E-01 | -7.27E-01 | 1.63E-02 | -1.30E-04 | 1.36E-03 | -4.94E-04 | 6.67E-02 | -7.23E-02 | 3.49E-06 |
|  | rcp85 | SR Rice | 3.19E+01 | -3.77E+01 | -6.07E-01 | 2.11E+00 | -1.15E-01 | 7.29E-04 | 8.26E-03 | -2.80E-03 | 6.10E-01 | 5.28E-01 | -4.16E-05 |
|  |  | LR Rice | 2.54E+01 | -3.67E+01 | -1.73E-01 | 4.86E+00 | -1.06E-01 | 6.55E-04 | 7.34E-04 | -2.13E-03 | 5.08E-01 | -9.12E-02 | -4.86E-06 |
|  |  | LR French beans | 2.59E+01 | -7.20E-01 | -2.37E-01 | -1.84E+00 | 2.21E-02 | -2.54E-04 | 2.12E-03 | 3.52E-04 | -2.95E-03 | 1.84E-01 | -7.82E-06 |
|  |  | LR Maize | 5.99E+00 | 7.40E+00 | -3.90E-01 | 2.65E+00 | 2.82E-02 | -2.54E-04 | 9.91E-03 | 2.67E-03 | -3.84E-01 | -9.00E-01 | -6.48E-05 |
|  |  | LR Soybeans | 2.87E+01 | 3.77E+00 | -4.99E-01 | -1.74E+00 | 2.30E-02 | -8.48E-05 | 9.80E-03 | -3.94E-04 | 3.49E-03 | 2.45E-01 | -4.79E-05 |
|  |  | LR Tomato | 2.58E+01 | -7.45E+00 | -2.68E-01 | -6.83E-01 | 1.72E-02 | -1.33E-04 | 7.83E-04 | -5.69E-04 | 7.70E-02 | -8.23E-02 | 9.95E-06 |
