# Supplementary material for: Integrating economic measures of adaptation effectiveness into climate change interventions: A case study of irrigation development in Mwea, Kenya
Source: PLoS One. 2020 Dec 11;15(12):e0243779. doi: 10.1371/journal.pone.0243779 (PMC7732349; doi:10.1371/journal.pone.0243779)
Supplement: S3 File — (DOCX) [file pone.0243779.s003.docx]

**S3 File.** **Estimated irrigation efficiency levels in the MIS (adapted from JICA and Nippon Koei 2018)**

(a) Without the irrigation development project

(b) With the irrigation development project
