# Supplementary material for: Integrating economic measures of adaptation effectiveness into climate change interventions: A case study of irrigation development in Mwea, Kenya
Source: PLoS One. 2020 Dec 11;15(12):e0243779. doi: 10.1371/journal.pone.0243779 (PMC7732349; doi:10.1371/journal.pone.0243779)
Supplement: S4 File — (DOCX) [file pone.0243779.s004.docx]

**S4 File.** **Estimated wholesale prices of crops**

| Commodity | Unit Price (Ksh/kg) | |
| --- | --- | --- |
|  | Baseline^*^ | 2030^**^, 2050^***^ |
| Rice (Basmati, short rain) | 45 | Upper bound: no change  Lower bound: 15% decrease |
| Rice (Basmati, short rain ratoon) | 33 |  |
| Rice (Basmati, long rain) | 60 |  |
| Dry maize | 41 | Upper bound: 10% increase  Lower bound: 10% decrease |
| Green gram | 103 |  |
| Tomato | 78 |  |
| Soybean | 60 |  |
| French bean | 31 |  |

Note: * According to the Rice Mapp 2016 survey (Basmati), the Ministry of Agriculture, Livestock and Fisheries (dry maize, green gram, tomatoes), the JICA internal study (Soybeans, French beans)

** Growth rates set to be the same as those of the October 2017 World Bank Commodities Price Forecast.

*** Set to be the same as the 2030 levels.
