# Supplementary material for: Integrating economic measures of adaptation effectiveness into climate change interventions: A case study of irrigation development in Mwea, Kenya
Source: PLoS One. 2020 Dec 11;15(12):e0243779. doi: 10.1371/journal.pone.0243779 (PMC7732349; doi:10.1371/journal.pone.0243779)
Supplement: Attachment — Submitted filename: PLOS ONE ARTICLE FOR REVIEW (PONE-D-20-19964_reviewer (1)) 1.pdf [file pone.0243779.s005.pdf]

PLOS ONE

Integrative Economic Evaluation of an Infrastructure Project as a Measure for Climate Change Adaptation: A Case Study of Irrigation Development in Kenya

--Manuscript Draft--

Manuscript Number:	PONE-D-20-19964
Article Type:	Research Article
Full Title:	Integrative Economic Evaluation of an Infrastructure Project as a Measure for Climate Change Adaptation: A Case Study of Irrigation Development in Kenya
Short Title:	Economic Evaluation of an Irrigation Development Project as a Measure for Climate Change Adaptation
Corresponding Author:	Daiju Narita, Ph.D.<br>University of Tokyo<br>Tokyo, JAPAN
Keywords:	climate change adaptation; economic assessment; irrigation; agriculture; runoff analysis; Africa
Abstract:	As climate change adaptation is becoming a recognized policy issue, the need is growing for quantitative economic evaluation of adaptation-related public investment, particularly in the context of climate finance. Irrigation, which enhances and stabilizes water supplies for farming, is a potential means of climate change adaptation, but attempts at economic evaluation of its effectiveness as an adaptation measure are few, in part because such assessments require an integration of various types of simulation analyses. Against this background, we conduct a case study of a Kenyan irrigation development project using a combination of simulation models to evaluate the effectiveness of that project for climate change adaptation. The results show that despite the uncertainties in precipitation trends, increased temperatures due to climate change have a general tendency to reduce rice yields, and that irrigation development will mitigate income impacts from the yield loss, i.e., will likely be effective as a means for climate change adaptation.
Order of Authors:	Daiju Narita, Ph.D.
	Ichiro Sato
	Daikichi Ogawada
	Akiko Matsuura
Additional Information:	
Question	Response
**Financial Disclosure**<br><br>Enter a financial disclosure statement that describes the sources of funding for the work included in this submission. Review the submission guidelines for detailed requirements. View published research articles from *PLOS ONE* for specific examples.<br><br>This statement is required for submission and **will appear in the published article** if the submission is accepted. Please make sure it is accurate.	This study is funded by JICA and the JICA Ogata Research Institute (JICA-RI), which also provided various logistical support. Daiju Narita acknowledges financial support from JSPS Kakenhi (Grant Numbers: 17KT0066 and 17K00677) for his research activities. Besides logistical support, the funders had no role in study design, data collection and analysis, decision to publish, or preparation of the manuscript.

Powered by Editorial Manager® and ProduXion Manager® from Aries Systems Corporation

**Unfunded studies**<br>Enter: *The author(s) received no specific funding for this work.*<br><br>**Funded studies**<br>Enter a statement with the following details:<br>• Initials of the authors who received each award<br>• Grant numbers awarded to each author<br>• The full name of each funder<br>• URL of each funder website<br>• Did the sponsors or funders play any role in the study design, data collection and analysis, decision to publish, or preparation of the manuscript?<br>• **NO** - Include this sentence at the end of your statement: *The funders had no role in study design, data collection and analysis, decision to publish, or preparation of the manuscript.*<br>• **YES** - Specify the role(s) played.<br><br>* typeset	
**Competing Interests**<br><br>Use the instructions below to enter a competing interest statement for this submission. On behalf of all authors, disclose any competing interests that could be perceived to bias this work—acknowledging all financial support and any other relevant financial or non-financial competing interests.<br><br>This statement **will appear in the published article** if the submission is accepted. Please make sure it is accurate. View published research articles from *PLOS ONE* for specific examples.	The authors have declared that no competing interests exist.

Powered by Editorial Manager® and ProduXion Manager® from Aries Systems Corporation

**NO authors have competing interests**<br><br>Enter: *The authors have declared that no competing interests exist.*<br><br>**Authors with competing interests**<br><br>Enter competing interest details beginning with this statement:<br><br>*I have read the journal's policy and the authors of this manuscript have the following competing interests: [insert competing interests here]*<br><br>* typeset	
**Ethics Statement**<br><br>Enter an ethics statement for this submission. This statement is required if the study involved:<br><br>• Human participants<br>• Human specimens or tissue<br>• Vertebrate animals or cephalopods<br>• Vertebrate embryos or tissues<br>• Field research<br><br>Write "N/A" if the submission does not require an ethics statement.<br><br>General guidance is provided below. Consult the submission guidelines for detailed instructions. **Make sure that all information entered here is included in the Methods section of the manuscript.**	N/A

Powered by Editorial Manager® and ProduXion Manager® from Aries Systems Corporation

**Format for specific study types**<br><br>**Human Subject Research (involving human participants and/or tissue)**<br>• Give the name of the institutional review board or ethics committee that approved the study<br>• Include the approval number and/or a statement indicating approval of this research<br>• Indicate the form of consent obtained (written/oral) or the reason that consent was not obtained (e.g. the data were analyzed anonymously)<br><br>**Animal Research (involving vertebrate animals, embryos or tissues)**<br>• Provide the name of the Institutional Animal Care and Use Committee (IACUC) or other relevant ethics board that reviewed the study protocol, and indicate whether they approved this research or granted a formal waiver of ethical approval<br>• Include an approval number if one was obtained<br>• If the study involved *non-human primates*, add *additional details* about animal welfare and steps taken to ameliorate suffering<br>• If anesthesia, euthanasia, or any kind of animal sacrifice is part of the study, include briefly which substances and/or methods were applied<br><br>**Field Research**<br><br>Include the following details if this study involves the collection of plant, animal, or other materials from a natural setting:<br>• Field permit number<br>• Name of the institution or relevant body that granted permission	
**Data Availability**<br><br>Authors are required to make all data underlying the findings described fully available, without restriction, and from the time of publication. PLOS allows rare exceptions to address legal and ethical concerns. See the PLOS Data Policy and FAQ for detailed information.	Yes - all data are fully available without restriction

Powered by Editorial Manager® and ProduXion Manager® from Aries Systems Corporation

A Data Availability Statement describing where the data can be found is required at submission. Your answers to this question constitute the Data Availability Statement and **will be published in the article**, if accepted.<br><br>**Important:** Stating 'data available on request from the author' is not sufficient. If your data are only available upon request, select 'No' for the first question and explain your exceptional situation in the text box.<br><br>Do the authors confirm that all data underlying the findings described in their manuscript are fully available without restriction?	
**Describe where the data may be found in full sentences. If you are copying our sample text, replace any instances of XXX with the appropriate details.**<br><br>• If the data are **held or will be held in a public repository**, include URLs, accession numbers or DOIs. If this information will only be available after acceptance, indicate this by ticking the box below. For example: *All XXX files are available from the XXX database (accession number(s) XXX, XXX.*).<br>• If the data are all contained **within the manuscript and/or Supporting Information files**, enter the following: *All relevant data are within the manuscript and its Supporting Information files.*<br>• If neither of these applies but you are able to provide **details of access elsewhere**, with or without limitations, please do so. For example:<br><br>*Data cannot be shared publicly because of [XXX]. Data are available from the XXX Institutional Data Access / Ethics Committee (contact via XXX) for researchers who meet the criteria for access to confidential data.*<br><br>*The data underlying the results presented in the study are available from (include the name of the third party*	Base data for the analysis are found in JICA and Nippon Koei (2018). Economic Evaluation of Adaptation Measures to Climate Change under Uncertainty Phase 2. Tokyo: JICA (in Japanese) -- which is mentioned in the reference list of the manuscript.

and contact information or URL).<br>• This text is appropriate if the data are owned by a third party and authors do not have permission to share the data.<br><br>* typeset	
Additional data availability information:	

Manuscript

Click here to access/download;Manuscript;Mwea_CCA_Jun2020

Integrative Economic Evaluation of an Infrastructure Project as a Measure for Climate Change Adaptation: A Case Study of Irrigation Development in Kenya

(Short title: Economic Evaluation of an Irrigation Development Project as a Measure for Climate Change Adaptation)

Daiju Narita[*1,2,3], Ichiro Sato[4], Daikichi Ogawada[5] and Akiko Matsumura[5]

1. Graduate School of Arts and Sciences, University of Tokyo, Tokyo, Japan

2. JICA Ogata Research Institute, Tokyo, Japan

3. Kiel Institute for the World Economy, Kiel, Germany

4. Japan International Cooperation Agency (JICA), Sendai, Japan

5. Nippon Koei Co., Ltd, Tsukuba, Japan

* Corresponding author
E-mail: daiju.narita@global.c.u-tokyo.ac.jp (DN)

Author contribution:
Conceptualization: DN and IS; Formal analysis: DO and AK; Methodology: all authors; Writing – Original Draft Preparation: DN; Writing – Review & Editing: all authors.

Integrative Economic Evaluation of an Infrastructure Project as a Measure for Climate Change Adaptation: A Case Study of Irrigation Development in Kenya

Abstract

As climate change adaptation is becoming a recognized policy issue, the need is growing for quantitative economic evaluation of adaptation-related public investment, particularly in the context of climate finance. Irrigation, which enhances and stabilizes water supplies for farming, is a potential means of climate change adaptation, but attempts at economic evaluation of its effectiveness as an adaptation measure are few, in part because such assessments require an integration of various types of simulation analyses. Against this background, we conduct a case study of a Kenyan irrigation development project using a combination of simulation models to evaluate the effectiveness of that project for climate change adaptation. The results show that despite the uncertainties in precipitation trends, increased temperatures due to climate change have a general tendency to reduce rice yields, and that irrigation development will mitigate income impacts from the yield loss, i.e., will likely be effective as a means for climate change adaptation.

Keywords:
climate change adaptation, economic assessment, irrigation, agriculture, runoff analysis, Africa

1. Introduction

As the effects of global climate change have become more evident in various parts of the world, the need for adaptation to these changes has become more apparent. Climate change adaptation necessitates public planning and investment concerning the provision of public goods (Mendelsohn 2012), and as such, there are growing demands for international assistance for adaptation actions in developing countries that are particularly vulnerable to the impact of climate change. UNEP-DTU (2016) estimates that annual adaption costs in developing countries will range from US$140 to 300 billion by 2030, which is two-to-three times higher than the previous estimates of future adaptation costs and will further increase to US$280-500 billion by 2050. Responding to such growing demands, international funds and institutions of development aid and finance intend to strengthen their support for adaptation action in those countries.

Against this background, the international climate and development community is starting to recognize the necessity for a methodology and metrics to quantitatively assess the effectiveness of climate change adaptation measures and to compare the adaptation effectiveness of different project options for desirable resource allocation and project planning. Such a methodology is required also to review the progress of and draw lessons from adaptation actions, and furthermore, it would ensure accountability for the resources

allocated to them. However, conventional methods of project evaluation are inadequate for such assessment because the impacts of climate change are highly uncertain due to the long time spans of these changes and a continuing lack of knowledge on the mechanisms of climate and other natural systems (e.g., Hallegatte et al. 2011; Burke et al. 2015). Indeed, an expert group of the United Nations Framework Convention on Climate Change (UNFCCC) indicates that the "nature of adaptation, including its long timescales, the uncertainty associated with its impacts and its context-specificity, and difficulties in setting baselines and targets and the consequent lack of common metrics to measure the reduction of vulnerability or the enhancement of adaptive capacity all constrain reviewing the adequacy and effectiveness of adaptation"[1].

Our study aims to respond to such methodological needs in the practice of climate change finance in terms of the economic evaluation of climate c[illegible]e adaptation-related projects subject to future uncertainty. To this end, we perform a quantitative case study evaluating an irrigation development project in Kenya. Our assessment is cross-discip[illegible]y in synthesizing modeling frameworks from climatology, hydrology, agronomy and decision science. The case was chosen as an example of climate change adaptation in general but has significance of its own. Africa is known to be one of the most vulnerable regions in terms of agriculture from climate change (Rosenzweig et al. 2014). At the same time, a large potential for irrigation exists in Africa and also in Kenya specifically (You et al. 2011, 2014), and promotion of irrigation could mitigate the negative effects of climate change in the region (Elliott et al. 2014).

Irrigation, which enhances and stabilizes water supply for farming, or makes farming "climate-proof," is a major means of climate change adaptation (Agrawala and Fankhauser 2008). Various global or regional-level assessments have already been made on climate change impacts and adaptation regarding African agriculture using process-based and statistical models (e.g., Seo et al. 2009; Schlenker and Roberts 2010; Müller et al. 2011; Rosenzweig et al. 2014; Adhikari et al. 2015; van Ittersum et al. 2016). Also, a number of survey-based economic studies have examined actual adaptation practices and perceptions in the face of the climate risks for African farmers (reviewed by Di Falco (2014)). However, to the authors' knowledge no economic studies of the assessment of climate change adaptation exist, in Africa or elsewhere, in the form of the project evaluation of irrigation development, despite the importance of such analysis in the context of climate finance as described above. Apart from practical usefulness, locality-specific analyses should also offer valuable insights for the general understanding of climate change adaptation as they can reflect realistic institutional arrangements and the particular socioeconomic and environmental conditions of those localities.

In principle, the effectiveness of an infrastructure project as a means of climate change adaptation should be evaluated as the avoided loss caused by climate change, expressed in a monetary unit[2] if it is to be integrated into the economic analysis of the project. A major

[1] Adaptation Committee and Least Developed Countries Expert Group, FCCC/SB/2017/2/Add.1–FCCC/SBI/2017/14/Add.1, dated in September 2017.

[2] Monetary-equivalent benefits could include both market and non-market benefits, the latter of which can be estimated by using a method of environmental valuation (stated or revealed preference methods).

difficulty in such an evaluation is the representation of uncertainty associated with climate change. In our study, we incorporate the uncertainties of future climatic and economic conditions by considering a large number of scenarios without any differentiation of their likelihood (i.e., no use of probabilistic weights), an approach consistent with the Robust Decision Making (RDM) method, a methodological framework of decision-making support under deep uncertainty (Lempert et al. 2013). This paper discusses the simulation methods and the basic results of our integrative modeling of irrigation impacts; a systematic evaluation of the impact of uncertainty on our simulation results based on the RDM framework will be presented in a different paper (Narita et al. in preparation). The results of our simulation analysis can directly be used as a cost-benefit assessment and thus is relatable to the concerns of the climate finance community mentioned above.

Our analysis reveals that climate change reduces crop yields and farmers' income in many of the scenarios considered, and an increase of water availability by irrigation development mitigates such negative impacts. In other words, development of irrigation infrastructure serves as a means of climate change adaptation. Meanwhile, it also shows the range of possible negative outcomes resulting from uncertainties is wider for cases without irrigation than with irrigation, indicating, among other things, the very large potential yield losses that could occur under climate change in the worst cases without new irrigation infrastructure.

The rest of the paper is organized as follows. Section 2 describes our study area and the target of this case study, the Mwea Irrigation Development Project in Kenya. Section 3 provides an outline of our evaluation approach, and Section 4 explains datasets used for analysis and simulation methods. The simulation results are shown and discussed in Section 5, while Section 6 concludes the paper.

2. The Study Area and the Mwea Irrigation Development Project

We conduct a case study of th[illegible]ea Irrigation Development Project~~, which~~ is an irrigation infrastructure development pro[illegible] in Mwea area of Kenya undertaken by Kenya's National Irrigation Board (NIB) with a loan provided by the Ja[illegible] International Cooperation Agency (JICA). See Figure 1 for a map of the Mwea area. The project consists of the construction of an irrigation dam (on the Thiba River, see Figure 1), the construction of a new irrigation canal (Link Canal III), and the improvement of existing canals and irrigation areas. Mwea is located approximately 100km northeast of Nairobi (Latitude 0˚41' S and Longitude 37˚20' E) at an elevation of 1160m above sea level. The local climate is tropical with two rainy seasons, a long rainy season from March to May and a short rainy season in October and November. Irrigation farming of rice and horticulture has extensively been carried out in the area since the 1950s by utilizing water from two local rivers, the Thiba and Nyamindi, and the irrigation system is currently managed under a public mechanism, the Mwea Irrigation Scheme (MIS). Households in the area predominantly engage in crop farming, andother industries are minor in the region. The present project is to increase the amount of available water for irrigation in the area. Although farming in Mwea has relied on irrigation water, it is carried out mostly only in the two rainy seasons as irrigation water is limited and needs to be supplemented by rainwater. This general limitation of irrigation water, along with local awareness of profitability of rice farming and demographic pressures, is reflected in local

interest in a new infrastructure project. With approximately 8,500ha of irrigated area and about 7,500 farming households, the MIS in its present form is by far the largest of the country's irrigation schemes and is where most rice production is undertaken in Kenya. Of the national irrigation schemes, the MIS accounts for about 80% of the total irrigated area and 90% of rice production (Short et al. 2012; Atera et al. 2016). Rice is the third most important cereal crop in Kenya after maize and wheat, and its consumption is rapidly growing. However, the vast majority of rice consumed in the country is imported, and the national government has set a long-term plan (the National Rice Development Strategy: NRDC) to increase rice production and thus reduce import dependency (Ministry of Agriculture, Livestock and Fisheries 2014).

The irrigation development project began its implementation process in 2010 and construction is ongoing as of February 2019. In the planning stage, climate change adaptation was not considered an explicit objective of the project, and thus prior to this study, the project's effectiveness as a measure of climate change adaptation has not been assessed. Although it did not focus on the climate change implications, a feasibility study was made by JICA in 2009 (JICA internal study for the Mwea Irrigation Development Project -- detailed information in this study report is given in JICA and Nippon Koei 2018). Alongside the irrigation development project, a technical cooperation project jointly funded by the Government of Kenya and JICA, called the Rice-based and Market-oriented Agriculture Promotion (Rice MAPP) Project, was also conducted in the Mwea between January 2012 and January 2017 (JICA 2017). The Rice MAPP was not a project specifically addressing climate change adaptation but demonstrated a number of farming practices potentially useful for climate change adaptation, such as water-saving techniques, the cultivation of alternative crop varieties, and the extended storage of harvested rice. The Rice MAPP ran economic surveys on farmers' socioeconomic status and on the local rice market, and along with the outcomes of the JICA internal study, these data are used as a basis for our case study.

3. Evaluation Approach

In this analysis, we evaluate the effectiveness of the irrigation development project on climate change adaptation defined as the difference in output quantities (e.g., yields) with and without the project under climate change relative to the difference of the same that would be expected in the absence of climate change (Figure 2). Non-irrigation uses of the water, such as power generation, drinking water supplies, and recreational use, are not considered in the project plan, and therefore their effects are not included in our analysis.

Economic metrics (income and others) are estimated by using a combination of simulation models, namely, climate, hydrological and yield forecasting models, which are soft-linked with each other. We do not build a single model including all these components but run computations by feeding the simulation outputs of each model to another. Figure 3 illustrates the flow of our simulation analysis. It shows that the change in climate influences the water balance, and that both of them serve as input for yield forecasting, which determines economic outcomes. Water balance analysis and economic analysis are

conducted by using the outputs of the three simulation models and also reflecting on the actual local conditions of cropping patterns, population, and so on.

Simulations are performed for a number of scenarios with varied climatic and socioeconomic parameters that represent the uncertainties for the Mwea (see Table 1 for the list of uncertainties considered). The scenarios are treated equally without probabilistic weights, as they are from different models rather than being probabilistic scenarios predicted by a single model – this treatment of uncertainty is consistent with the RDM framework as mentioned above. It should be noted that the primary focus of our uncertainty analysis is to identify system behavior in the face of possible changes in key conditions, which local stakeholders are interested in (see the next paragraph). In other words, we are not concerned with the nature of the uncertainties. Hence, in the simulations we do not distinguish the treatment of uncertainties from different sources, such as natural randomness and incomplete knowledge, as long as the local stakeholders have no control over them.

To reflect local concerns about climate risks in the model simulations, we carried out our analysis in two stages – preliminary and main – holding a set of stakeholder interviews in between. In May 2017 we held meetings with officials and representatives of the following organizations, presenting to them the preliminary results from our analyses, and obtaining feedback: the Mwea Irrigation Agricultural Development Centre (MIAD), the National Irrigation Board (NIB), the Irrigation Water Users Association (IWUA), the Ministry of Water and Irrigation, the Ministry of Agriculture, Livestock and Fisheries, and the Kenya Meteorological Department. We subsequently conducted the main analysis by reflecting their comments, and this paper reports on the results of the main analysis.

4. Data and Simulation Methods

In this section, we describe the data [illegible] simulation methods relating to climate, hydrology, water balance, yields and economic outcomes. The modeling frameworks of runoff analysis and yield forecasting are built on those used by the JICA internal study mentioned above, and part of the data for calibration are drawn from the report (see JICA and Nippon Koei 2018 for details). We use standard, publicly-available data and models for the simulation of climatic conditions, yields and economic outcomes. This means that our assessment approach could be applied to many other cases of irrigation development, including those in relatively data-scarce developing countries.

4.1. Development of Climate Scenarios

We develop scenarios of future climatic conditions in Mwea by using a simplified method of downscaling. The downscaling method we use for this analysis is a version of the delta change method as employed by Prudomme et al. (2010). In this method, changes of climatic variables (in our case, precipitation and temperature) are calculated as the differences in the output values of global circulation models (GCMs) for the baseline (current) and future periods on the model grid encompassing the target location. Then the estimated changes

are added or multiplied to the observational weather levels at the baseline period. More precisely, future levels of climatic variables are computed according to the following formula for a climate variable *X*:

$$\tilde{X}_{iy,im,j}^{GCM\_fut} = CF_{im} \cdot X_{iy,im,j}^{OBS\_hist} \qquad \text{(for precipitation)} \tag{1}$$

or,

$$\tilde{X}_{iy,im,j}^{GCM\_fut} = CF_{im} + X_{iy,im,j}^{OBS\_hist} \qquad \text{(for temperature)} \tag{1'}$$

Where: *iy*, *im*, and *j* are indices for the year, the month and the day of the month, and a tilda (as in $\tilde{X}$) denotes the projected future value. A *CF* is the change factor, which is defined as the absolute (for temperature) or percentage (for precipitation) change in the means between the baseline and future time-slices.

The GCM data used for these estimations are obtained from the CMIP5 (Coupled Model Intercomparison Project Phase 5) database, which is publicly available and contains simulation results of a number of GCMs used for the IPCC AR5 report. It [illegible]d not have been appropriate, however, that we use data from all models indiscrimi[illegible]y for our purpose because some of these global models grossly misrepresent the specific climatic conditions of Kenya. Hence, for our dataset, we screen the available 47 GCMs in the dataset by applying the Interquartile Range Rule to temporal and spatial correlations and root mean square errors (RMSEs) of the model outputs for the present (evaluated for the area 10˚S-10˚N, 25˚E-50˚E). We select the outputs of 14 models that have passed this screening, each of which has datasets for 4 RCP (Representative Concentration Pathway) scenarios (RCP 2.6, RCP 4.5, RCP 6.0, and RCP 8.5: van Vuuren et al. 2011). Differences in RCP scenarios lead not only to different predicted future climatic conditions but also to differences in the carbon dioxide fertilization effect, which influences crop yields (see also Section 4.3). Note that from the standpoint of local stakeholders in Mwea, the global emission paths are basically fully given rather than subject to their control, and thus the simulation analysis does not distinguish the scenario differences of different GCMs and different RCPs.

In addition to the GCM data, observational weather data are needed for the application of the delta change method mentioned above. For Mwea, some observational local weather data exist but have frequent interruptions. Therefore, we primarily use the WFEDI (WATCH-Forcing-Data-ERA-Interim) reanalysis data (Weedon et al. 2014: 0.5˚ x 0.5˚ resolution), a global dataset of model-interpolated observational weather data, for the delta-change adjustment of GCM-based values.

Since farming practices in Mwea are sensitive to the seasonality of precipitation, estimation of future changes in the seasonal patterns of rainfall (especially the onset of the rainy seasons) could greatly affect our results. We therefore consider future changes in rainfall seasonality by representing the seasonal variation in a functional form. Estimated future changes in monthly values of precipitation from each GCM are fitted to a harmonic function with one node, which is characterized by amplitude (C_1) and phase (φ_1) parameters as well

as shifts in the annual average (X_0). The ranges in the levels of these parameters and of shifts in annual averages of temperature[3] are considered the ranges in our case sampling (as described below). As an alternative set, we also generate scenarios ignoring the change in precipitation seasonality. We do not conduct a fitting with a harmonic function for the seasonality of temperatures since seasonal variations of temperatures are relatively small, and play in the context of Kenya a relatively minor role in farming.

The change factor *CF* is computed according to the following formulae:

(2) $$CF_{im} = X_0 + C_1 \cdot \cos(\frac{2\pi \cdot im}{12} - \varphi_1)$$ (for precipitation)

(2') $$CF_{im} = X_0$$ (for temperature)

where *im* is the index for the month.

For each climate scenario, we set up a spatial dataset (with 0.1˚ x 0.1˚ grids for the region 0.9˚S-0.1˚S, 37.1˚E-37.6˚E) of the following weather variables for three time periods, the present/historical (average over the period 1991-2010), the reference year of 2030 (average over the period 2021-2040), and the reference year of 2050 (average over the period 2041-2060). The variables are precipitation, temperature (daily average, maximum and minimum), surface wind speed, relative humidity, shortwave radiation, and surface air pressure. The last four weather variables are necessary for calculating evapotranspiration (i.e., the reference evapotranspiration ET_0 by the FAO Penman-Monteith method), which is an input for the hydrological model described below (Section 4.2). For these four variables, we use the values of present observational data (WFEDI reanalysis data) for all the three modeling periods by assuming that the levels will remain unchanged in the future.

Since one of the main objectives of our analysis is to assess the sensitivity of outcome variables to uncertainties of plausible climate conditions and parameter levels in the future, we do not use the above-described estimates of future climate from the 14 models directly but rather construct climate scenarios by using the following method of random case generation. We first identify the upper and lower bounds in the shifts in the levels of the weather variables among the constructed climate data of the 14 models and then preform a Latin Hypercube Sampling (LHS) from the identified ranges to generate a set of scenarios (60 scenarios). Figure 4 shows our estimated changes in annual average temperature and precipitation for 20[illegible]nd 2050 from the baseline levels under different RCP scenarios, represented in a range (i.e., the highest and lowest values of model-derived estimates). The graphs exhibit a clear tendency for increasing temperatures over the years, by around 1˚C in 2030 and somewhat greater than that level in 2050. On the other hand, the future changes in precipitation are unclear even in sign, although the ranges tend to be skewed in the positive direction (i.e., greater amounts of precipitation).

4.2. Runoff Analysis and Water Balance Analysis

[3] More precisely, the daily maximum and minimum temperatures. Although the two variables are different, their changes relative to those in the current climate show nearly identical patterns.

The amounts of available irrigation water in Mwea are modeled in a semi-empirical fashion by using a physical hydrological model that covers the catchment areas of Thiba and Nyamindi Rivers,[4] and calibrating this with the relationship between the constructed weather data from the WFDFI and the observational data of river flows at two monitoring stations in the Mwea area (Thiba and Rupingazi stations) during the period 1981 to 2010. This is the same approach to hydrological modeling as that taken by the JICA internal study in 2009, but its calibration is renewed for this study and new simulations of conditions under climate change are now performed. The calibrated model is used to compute river flows and water distribution across the farming areas in the MIS, while applying allocation rules consistent with present farming practices and water rights under the MIS (details on this are discussed in JICA and Nippon Koei 2018). For the simulations of hydrological processes, we use the SHER (Similar Hydrologic Element Response) model, which is a hydrological model originally developed by Herath et al. (1990) and applied in a wide range of evaluations of water resource related projects, including the estimations for the Kenyan National Water Master Plan 2030 published in 2014[5]. The SHER model is a physical model that represents the hydrology of a watershed by a set of simple physical equations with parameters (porosity and conductivity of soil, and so on), whose levels are empirically set by using observational data and the results of laboratory testing. The model simulates hydrological processes by differentiating between recharging and discharging areas, the latter being the surroundings of Mwea in our case study, and the distinction of these two types of areas is made manually by the analyst. The version of SHER we use covers the catchment areas of Thiba and Nyamindi Rivers from Mount Kenya (upstream) to the Mwea area and is composed of sub-models of surface flows, subsurface flows, and aquifers. For parameterization, it utilizes publicly available datasets of soil type and land use: for soil type data, the KENSOTER (Kenya Soil and Terrain database, version 2.0) by the Kenya Soil Survey (KSS) and ISRIC, and for land use data the Africover database by the FAO. A detailed model description is given in the Water Master Plan document and the JICA internal study.

The available amounts of water for irrigation in Mwea are estimated as the differences in the modeled river flow rates at the three water intake points of the MIS (the New Nyamindi Headwork, Thiba Dam, and Thiba Headwork, all shown in Figure 1) and the amounts of the minimum required flows rates, i.e., the total amounts of the existing water rights in the areas downstream from the three points plus the minimum amounts of water flows necessary to keep the integrity of the river systems. Following the JICA internal study, we set the minimum required flow rates at the New Nyamindi Headwork, Thiba dam and Thiba Headwork at 0.88, 0.98, and 1.86m^3/s, respectively. Precipitation and water flow data are available on a daily basis, but we only used monthly averages for analysis as our methodological approach does not yield reliable estimates of extreme weather values. However, since the target of our analysis is irrigation farming whose objective is to smooth out water inputs for crops, the omission of day-to-day fluctuations of precipitation does not greatly influence modeling results.

Figure 5 shows the mean river flow rates at the New Nyamindi Headwork under different climate scenarios, represented as scatterplots of mean flow levels in four seasons (the

[4] No glaciers exist in the catchment areas of these rivers.
[5] This document is available at https://wasreb.go.ke/national-water-master-plan-2030/

upper graphs are for Oct-Nov-Dec and Mar-Apr-May, and the lower graphs are for Jun-Jul-Aug and Jan-Feb). This presents two sets of results, one for 2030 and the other for 2050. The black dots correspond to the baseline levels, and the blue and yellow circles represent the estimates without and with seasonality adjustments by approximation with a harmonic function. The graphs indicate a general increase and decrease in river flows in Mar-Apr-May and Jun-Jul-Aug, respectively. In other words, in the vicinity of Mwea, climate change tends to magnify river flows in the long rainy season and make water scarcity acute in the dry months of June to August. Meanwhile, the trends in water quantities in the other seasons are unclear. Also, results for 2030 and 2050 are very similar, except that the latter exhibit a somewhat greater variance than the former. Adjustments of seasonality with a harmonic function generally yields greater river flows in Mar-Apr-May and Jan-Feb.

Collected water at the Headworks is distributed to farmlands through irrigation channels, which are subject to irrigation efficiency rates reflecting water leakage (See Appendix 3). We simulate water allocations across farmlands that are made proportional to the actual existing water rights, in other words, they may not be optimal distributions to maximize regional crop production (the possibility that a better water allocation might raise the farming productivity is not considered, given the reality of actual water management in the MIS). In the current farming practices of Mwea, a substantial number of farmers are utilizing irrigation water without official water rights (they are called "out growers" in the area). In the simulations, despite their informal status, we assume that these farmers will use water in the same way as the entitled farmers. Water supply affects yields and economic outcomes through influencing the sufficiency rate of water for crop farming, i.e., reduced water distributions lead to less than optimal water inputs for individual croplands and consequently result in a decline in yields. Seasonal water demand depends on the growth of cultivated crops and local cropping patterns, which are described in Sections 4.3 and 4.4.

4.3. Yield Forecasting

For yield simulations, we construct impact functions of crop yields for changes in climatic variables from the baseline conditions of Mwea by approximating the outputs of DSSAT (Decision Support System for Agrotechnology Transfer) model simulations for Mwea farming[6] into polynomial functions of temperature and water input. We use functional approximations of DSSAT rather than the model directly, for the reasons of favoring transparency in our modeling assumptions and also of managing the practical needs of computing numerous scenarios. The functions are built for rice (Basmati 370, the rice variety mainly grown in Mwea) from ordinary paddy production and ratoon (the second shoot from the base of a rice plant after cropping) production, and for five major upland crops (maize, tomato, green gram, French beans, and soybeans). Yield impacts are represented as the percentage change in yield from the baseline level, and functional forms are differentiated for rice production in the short and long rainy seasons. Specific forms of these functions are given in Appendix 2.

[6] For simulations, we used the DSSAT version 4.6 released in 2014.

DSSAT is a software program consisting of 40 crop models[7] (Jones et al. 2003) and is applied in a wide range of crop simulation analyses worldwide. Its crop models compute yields and other variables from the input parameters of weather conditions, soil type and conditions, and crop management. In our modeling context, the main advantage of using DSSAT over other crop simulation models is that it has already been applied in the analysis of Mwea rice farming (Nyang'au et al. 2014), and thus information about parameter calibration is partly available. However, DSSAT does not have parameter sets for one of the upland crops we consider (green gram) and for the exact varieties of the other crops grown in Mwea. For these, we use the parameter sets of DSSAT for a similar crop (soybeans for green gram) or similar varieties (for the upland crops other than green gram) and adjust the yield output by using the actual yield data in Mwea given in the JICA internal study report. Also, ratoon production is not explicitly considered in the DSSAT framework, for our simulations of ratoon cultivation we used the DSSAT simulation results for ordinary paddy rice and applied to them the yield ratio of ordinary paddy production and ratoon production estimated in the JICA internal study (0.45).

By interpolating model outputs from DSSAT simulations, in the setting of Mwea we set up yield functions of the two most influential parameters on yields, seasonal mean daily average temperature and water input. For most of the other parameters for model calculation, such as the timing and amount of fertilizer input, we set the levels to achieve maximal production under most scenarios. Meanwhile, the setting of planting dates, which influences yield results, is made consistent with those considered in the JICA internal study and the RiceMAPP study (i.e., the dates are not optimally set to maximize yield values in DSSAT).

Yields are estimated separately for each of the considered RCP scenarios (RCP 2.6, RCP 4.5, RCP 6.0, and RCP 8.5), to reflect different levels of atmospheric carbon dioxide concentrations across these scenarios and consequently the different extent of the carbon dioxide fertilization effect, which partly offsets yield losses from temperature increases (DSSAT can estimate the carbon dioxide fertilization effect). Note that the scale of the carbon dioxide fertilization effect in yield forecasting models is generally subject to large uncertainties, and for this reason, some modeling studies exclude this effect in simulations (see for example OECD 2015). In this sense, our loss estimates could be seen as being relatively conservative (i.e., small). In parallel with yield computations, we estimate water demand for crops (crop coefficients K_c by the Penman-Monteith method) at each stage of plant growth based on FAO guidelines (Doorenbos and Pruitt 1977) and use this information for the water balance analysis discussed above (Section 4.2). Appendix 1 shows our estimates of crop coefficients. Here again, despite the fact that DSSAT can compute water demand, we do not use DSSAT directly for that purpose in favor of the transparency of our simulation assumptions.

4.4. Economic Analysis

[7] For estimations of rice yields we used the CERES Rice Model among the models embodied in DSSAT.

Economic analysis is carried out for a hypothetical case without an irrigation dam ("donothing") and for four options of possible cropping patterns after the irrigation development project is completed, namely "RiceRice," "RiceUpland," "RiceRice+," and "RiceUpland+," which differ in crops grown in the long and short rainy seasons and in the adoption of improved farming practices and techniques proposed by the Rice MAPP project. These practices include the following: the Water Saving Rice Culture (WSRC), a set of water saving techniques for rice farming; the Improved Ratoon Production (IRaP), improved practices of water and fertilizer applications to raise yields of ratoon farming[8] ; the Warehouse Receipt System, the storage of harvested rice to sell it in the peak season of demand; and mechanization of farming[9]. See Table 2 for a description of the options.

There are no data about the current scale of farming of upland crops in Mwea, and in our analysis, we assumed equal land areas allocated for each of the five major upland crops (maize, tomato, green gram, French beans, soybeans) considered in the options RiceUpland and RiceUpland+. In principle, farmers can maximize their profits by changing the shares of cultivated crops. For example, growing tomatoes is highly profitable in Mwea, and thus a shift from rice farming to tomato farming should generally raise farmers' income. However, a number of determinants other than profit exist in the real world context of local farming -- for example, tomatoes are relatively easily perishable, and thus in the weak logistic system of Mwea a large quantity of tomatoes cannot be reliably delivered to major markets, not to mention the general high revenue volatility resulting from the farming of fresh vegetables. For this reason, while in our analysis we contrast scenarios of enhanced rice farming and farming emphasizing upland crops, we do not perform a detailed analysis of the varied compositions of cultivated crops in this study.

We estimate indices of economic outcomes for combinations of climate and socioeconomic scenarios listed in Table 1. For the socioeconomic scenarios, we generate 100 sets of these by an LHS method[10], which randomly selects combinations of the values of socioeconomic parameters whose possible levels are assumed to be distributed uniformly between given upper and lower bounds.

Among the parameters of socioeconomic scenarios, the size of population in Mwea (MIS) is a parameter affecting the household income. We set the baseline number of households at 7,453 according to a survey made in 2016 by the Rice MAPP project (JICA 2017). In the years 2030 and 2050 we consider the lower bound of population to be the current level (given the water limitations in Mwea). The upper bound is set to be consistent with the estimates of UN projections of rural population in Kenya, i.e., a 47 % increase in 2030 and a 125% increase in 2050 (See Table 1). With the current irrigated area of 8,362ha (it will remain the same after the irrigation development project), this means that each farming household will have about 1ha, or 2.5 acres, with no population increase, and half that area with a doubled population. As identified by the survey carried out for the JICA internal study, although farmlands are not private properties in an official sense, they are mostly transferred to

[8] Based on the RiceMAPP data, we set the yield increase effect of WSRC and IRaP to be 13% and 17%, respectively, and the water saving effect of WSRC to be 20% of water input.
[9] Based on the RiceMAPP data, we assumed that mechanization reduces the production costs by 10%, water input by 30%, and the harvest loss by 6 percentage points.
[10] We computed LHS using the pyDOE package of Python.

junior family members upon a scheme commissioner's consent when the head of an MIS household dies. Given the high population growth rates of Kenya including the Mwea area, it is likely therefore `that farmlands in Mwea will remain settled by family members of the existing farmers in the future (i.e., an inflow of new farmers from outside is unlikely).

For the evaluation of farmers' income, we use wholesale crop prices estimated as in Appendix 4.[11] The baseline crop prices are set based on the data from the following three sources: the Rice MAPP survey in 2016 (JICA 2017), the National Farmers Information Service (NAFIS: www.nafis.go.ke) by the Ministry of Agriculture, Livestock and Fisheries, and the JICA internal study. We then estimate the 2030 prices by applying the growth rates of the world commodity prices forecast by World Bank Commodities Price Forecast (as of October 26 2017)[12] to these baseline prices, and we assume no systematic trend of prices from 2030 to 2050. The base year of currency units is the year 2010. As crop farming is the dominant source of income for the farmers in the MIS (which is identified by, for example, the Rice MAPP survey: JICA 2017), farmers' income is evaluated as the sales revenues from crops at wholesale prices minus the production costs estimated by the JICA internal study.

Along with the above analysis, we also compute the conventional metrics of cost-benefit analysis such as the net present value (NPV) for the irrigation development project, reflecting climate change and uncertainties (Appendix 5 shows a selected result). To this end, multiple possibilities of the social discount rate (values randomly chosen between 5 percent/yr and 10 percent/yr) are also considered in the LHS sampling.

5. Results of the Simulation Analysis

Figure 6 shows the box plots of simulation results of farmers' annual income and the total rice production of Mwea (MIS) for the four options in the irrigation development project and for the case without the irrigation development project ("donothing"). Each plot represents income level under one of 24,000 scenarios in Figure 6(a), or rice yield under one of 240 scenarios for Figure 6(b) (see also Table 1), in other words, their distributions represent uncertainties. The ends and middle lines of the boxes correspond to the quantiles (i.e., the middle line represents the median), and the whiskers represent the 1.5 interquartile ranges (IQRs). The Figure shows that estimated income levels for the four options with the irrigation development project are generally higher than those without the irrigation development project, although some overlaps in the range of boxes and whiskers exist. Adoption of improved farming techniques also leads to generally higher levels of income relative to the cases without them (identified through comparison between RiceRice and RiceRice+, and comparison between RiceUpland and RiceUpland+), while the rice yields for the options involving double-cropping of rice (RiceRice and RiceRice+) naturally exhibit higher levels of rice production than those with the farming of upland crops (RiceUpland and RiceUpland+). Without the irrigation development project ("donothing"), farmers'

[11] The use of wholesale prices is inappropriate for the cost-benefit assessment of the project based on NPV, EIRR, etc (see below). We have thus also developed an alternative set of crop prices for evaluation based on the world prices of crops in the World Bank Commodities Price Forecast.

[12] http://pubdocs.worldbank.org/en/678421508960789762/CMO-October-2017-Forecasts.pdf (last accessed 28 January 2018).

income is reduced in the future periods in all scenarios, while the total rice production generally turns negative from 2030 to 2050. For the four options with the irrigation development project (RiceRice, RiceUpland, RiceRice+, RiceUpland+), results for the reference years 2030 and 2050 are mostly similar, except that the variance of results becomes somewhat magnified from 2030 to 2050.

The effects of climate change are made more visible in Figure 7, which isolates the relative changes of rice yields (Graph a) and average household income (Graph b) in Mwea from the baseline levels that assume no climate change (see Figure 2 for the illustration of this evaluation). The results are shown as percentage changes from the baselines. For both graphs, the dotted horizontal line corresponds to zero change (no effects of climate change), and the boxes represent changes from the baseline under different climate and socioeconomic scenarios. Overall, the plots indicate great uncertainties about climate change impacts, with many areas both below and above zero for all the five sets of results. However, especially for 2050, the general tendency of negative yield changes under climate change is clear (recognizable by, for example, the fact that the median is negative) – this is a noteworthy result given that the predicted trends of precipitation are ambiguous, in other words, elevated temperatures alone could cause yield losses. In 2050, the yield and income losses caused by climate change are most significant for the case without the irrigation development project (donothing), while this tendency is not clear for the year 2030 (in fact, a majority of the donothing results for 2030 exhibit positive changes in yield and income). At least for 2050, relative to the donothing results, the distributions for the other four options are generally higher both in yield and income, and many scenarios of the four options with irrigation development exhibit positive income changes due to climate change. These results are an indication that the irrigation development project mitigates [illegible]negative effects of climate change, in other words, it serves as an effective means of climate change adaptation. This is consistent with the result shown in Appendix 5 that project benefits are generally enhanced by the inclusion of climate change impact[illegible]he evaluation. It is also noticeable in Figure 7 that the spread of boxes is greater in 20[illegible]an in 2030, corresponding to the greater uncertainty of climate change in the former year than in the latter year. The worst possible outcome for the donothing case for 2050 is a greater than 50% loss in income which would be mitigated by the irrigation development[illegible]ect.

This general finding on climate change impact, i.e., the trend of significant negative effects of climate change on agriculture, is broadly consistent with the findings of existing studies dealing with climate change impacts on agriculture in Africa, such as Seo et al. (2009), Müller et al (2011), and Rosenzweig et al. (2014). Also, our results are in line with the conclusion of Lobell and Burke (2008), in that temperature changes are more important for crop yields than precipitation changes, and thus that despite the inconclusiveness of precipitation trends in the target area under climate change, robust negative effects of climate change are predicted because of the unambiguously increasing trends of temperature alone.

7. Conclusion

As climate change adaptation is becoming a prominent global issue, the need for evaluation methodologies for adaptation-related public investments that reflect the uncertainties of climate change are growing, especially in the field of climate finance. This study sought to respond to such methodological interests through a case study of a Kenyan irrigation development project. We evaluate the effectiveness of the project on climate change adaptation, inclusive of the uncertainties in climate change and socioeconomic factors. Our simulation results show that despite the uncertainties in precipitation trends, the higher temperatures resulting from climate change will have a general tendency to reduce farmers' income due to lower crop yields, and that irrigation development will mitigate that income loss, i.e., it will likely function as a means of climate change adaptation. The results also indicate that irrigation could mitigate the worst possible outcomes of yield and income loss under climate change that would be realized without such irrigation development projects.

The general finding of our study that significant impacts of climate change on agriculture are expected in as early as a decade ahead is consistent with findings by global and regional-level studies of process-based and statistical models dealing with climate change impact on agriculture in Africa such as Seo et al. (2009), Müller et al. (2011), and Rosenzweig et al. (2014). Unlike some of these macro-level studies, our case study does not examine general equilibrium effects and does not analyze the optimality of agricultural practices. In return, however, our case-based approach can reflect realistic local institutional arrangements and circumstances, e.g., the local needs for the support of an increasing number of farmers in response to rapid rural population growth, not to mention that it can also incorporate detailed local characteristics of climate, hydrology, agronomy, and socioeconomic conditions. Therefore, we also stress that further case- or project-based insights of climate change adaptation are ultimately needed in the context of climate finance and project appraisal of public investments.

Acknowledgments

This study is funded by JICA and the JICA Ogata Research Institute (JICA-RI), which also provided various logistical support. We benefited from discussions with and inputs by the following people: Ichiro Adachi, Kotaro Taniguchi, Hiroshi Takeuchi, Daigo Makihara, Yuji Masutomi, Kiyoshi Takahashi, Koji Dairaku, Wycliff Nyang'au, Martin Gómez-Garcia, Julie Rozenberg, Laura Bonzanigo, Stephane Hallegatte, and Marianne Fay. Seminar and meeting participants at the following venues and institutions provided us with valuable insights: JICA, JICA-RI, the National Institute for Environmental Studies, the JpGU Meeting 2018, the 2019 AGU Fall Meeting, and the Kenyan institutions mentioned in the main text. Daiju Narita acknowledges financial support from JSPS Kakenhi (Grant Numbers: 17KT0066 and 17K00677) for his research activities.

References

Adhikari, U., A. P. Nejadhashemi & S. A. Woznicki (2015). "Climate change and eastern Africa: a review of impact on major crops." *Food and Energy Security* 4(2): 110–132.

Agrawala, S. & S. Fankhauser, eds. (2008). *Economic Aspects of Adaptation to Climate Change: Costs, Benefits, and Policy Instruments*. Paris: OECD.

Atera, E. A., F. N. Onyancha & E. O. Majiwa (2018). "Production and marketing of rice in Kenya: Challenges and opportunities." *Journal of Development and Agricultural Economics* 10(3): 64-70.

Burke, M., J. Dykema, D. B. Lobell, E. Miguel & S. Satyanath (2015). "Incorporate climate uncertainty into estimates of climate change impacts." *Review of Economics and Statistics* 97(2): 461–471.

Cervigni, R., R. Loden, J. E. Neumann & K. M. Srzepek, eds. (2015). *Enhancing the climate resilience of Africa's infrastructure*. Washington DC: World Bank.

Di Falco, S. (2014). "Adaptation to climate change in Sub-Saharan agriculture: Assessing the Evidence and Rethinking the Drivers." *European Review of Agricultural Economics* 41 (3): 405–430.

Doorenbos, J. & W.O. Pruitt (1977). *Guidelines for Predicting Crop Water Requirements*. FAO Irrigation and Drainage Paper 24. Rome: FAO.

Elliott, J., et al. (2014). "Constraints and potentials of future irrigation water availability on agricultural production under climate change." *PNAS* 111(9): 3239–3244.

Hallegatte, S., A. Shah, R. Lempert, C. Brown & S. Gill. (2012). *Investment decision making under deep uncertainty: Application to climate change*. Policy Research Working Paper No. 6193. Washington DC: World Bank.

Herath S., N. Hirose & K. Musiake (1990). “A computer package for the estimation infiltration capacities of shallow infiltration facilities.” In the *Proceedings of the 5th International Conference on Urban Storm Drainage*, Japan, pp.111-118.

JICA (2017). *Terminal evaluation report of the Rice-based and Market-oriented Agriculture Promotion Project (Rice MAPP)*. Tokyo: JICA (in Japanese).

JICA and Nippon Koei (2018). *Economic Evaluation of Adaptation Measures to Climate Change under Uncertainty Phase 2*. Tokyo: JICA (in Japanese).

Jones, J. W., G. Hoogenboom, C. H. Porter, K. J. Boote, W. D. Batchelor, L. A. Hunt, P. W. Wilkens, U. Singh, A. J. Gijsman, and J. T. Ritchie. 2003. “DSSAT Cropping System Model.” *European Journal of Agronomy* 18:235-265.

Kenya Ministry of Agriculture, Livestock and Fisheries. 2014. *National Rice Development Strategy (2008-2018)*. Revised Edition. Nairobi: Ministry of Agriculture, Livestock and Fisheries.

Lempert, R., N. Kalra, S. Peyraud, Z. Mao, S. B. Tan, D. Cira & A. Lotsch (2013). *Ensuring robust flood risk management in Ho Chi Minh City*. Policy Research Working Paper No. 6456. Washington DC: World Bank.

Lobell, D. B. & M. B. Burke (2008). “Why are agricultural impacts of climate change so uncertain? The importance of temperature relative to precipitation.” *Environmental Research Letters* 3: 034007.

Mendelsohn, R. (2012). “The economics of adaptation to climate change in developing countries.” *Climate Change Economics* 3(2): 1250006.

Müller, C., W. Cramer, W. L. Hare & H. Lotze-Campena (2011). “Climate change risks for African agriculture.” *PNAS* 108(11): 4313–4315.

Nyang’au, W. O., B. M. Mati, K. Kalamwa, R. K. Wanjogu & L. K . Kiplagat (2014). “Estimating Rice yield under changing weather conditions in Kenya using CERES rice model.” *International Journal of Agronomy* Article ID 849496. doi:10.1155/2014/849496.

OECD (2015). *The Economic Consequences of Climate Change*. Paris: OECD.

Prudomme, C., R. L. Wilby, S. Crooks, A. L. Kay & N. S. Reynard (2010). “Scenario-neutral approach to climate change impacts: Application to flood risk.” *Journal of Hydrology* 390: 198-209.

Rosenzweig, C. et al. (2014). “Assessing agricultural risks of climate change in the 21st century in a global gridded crop model intercomparison.” *PNAS* 111(9): 3268–3273.

Schlenker, W. & D. B. Lobell (2010). “Robust negative impacts of climate change on African agriculture.” *Environmental Research Letters* 5: 014010.

Seo, S. N., R. Mendelsohn, A. Dinar, R. Hassan & P. Kurukulasuriya (2009). "A Ricardian Analysis of the Distribution of Climate Change Impacts on Agriculture across Agro-Ecological Zones in Africa.” *Environmental and Resource Economics* 43:313–332.

Short, C., W. Mulinge & M. Witwer (2012). *Analysis of incentives and disincentives for rice in Kenya*. Technical notes series, MAFAP. Rome: FAO.

UNEP-DTU (2016). *Adaptation Gap Report 2016.* Copenhagen: UNEP-DTU.

United Nations (2017). *World Population Prospects: The 2017 Revision*. New York: UN.

van Ittersum, M. K., et al. 2016. “Can sub-Saharan Africa feed itself?” *PNAS* 113 (52): 14964-14969.

van Vuuren, D. P., J. Edmonds, M. Kainuma, et al. (2011). “The representative concentration pathways: an overview“ *Climatic Change* 109: 5–31.

Weedon, G. P., G. Balsamo, N. Bellouin, S. Gomes, M. J. Best & P. Viterbo (2014). “The WFDEI meteorological forcing data set: WATCH Forcing Data methodology applied to ERA-Interim reanalysis data.” *Water Resources Research* 50. doi:10.1002/2014WR015638.

You, L., C. Ringler, U. Wood-Sichra, R. Robertson, S. Wood, T. Zhu, G. Nelson, Z. Guo & Y. Sun (2011). “What is the irrigation potential for Africa? A combined biophysical and socioeconomic approach.” *Food Policy* 36: 770–782.

You, L., H. Xie, U. Wood-Sichra, Z. Guo & L. Wang (2014). “Irrigation potential and investment return in Kenya.” *Food Policy* 47: 34–45.

Table 1. Types and number of scenarios (uncertainties) considered in the analysis

	Type of uncertainty	Number of scenarios	Note
Climate scenarios	RCPs (CO_2 concentration)	4	RCP 2.6, RCP 4.5, RCP 6.0, RCP 8.5
	Temperature changes	60<br>(LHS sampling)	Sampling range set by outputs of 14 GCMs
	Precipitation changes		
	Seasonality change of precipitation		
	(Subtotal)	(240)	
Socio-economic scenarios	Household number in MIS	100<br>(LHS sampling)	In 2030<br>Upper bound: 47% increase<br>Lower bound: no increase<br><br>In 2050<br>Upper bound: 125% increase<br>Lower bound: no increase
	Price of rice		See Appendix 4 for specifications<br><br>Upper bound: no change<br>Lower bound: 15% decrease
	Price of upland crops		Upper bound: 10% increase<br>Lower bound: 10% decrease
	Production cost		Upper bound: 30% increase<br>Lower bound: 30% decrease
	Discount rate		Upper bound: 10%/yr<br>Lower bound: 5%/yr
		24,000	

Table 2. Options of cropping patterns and improved farming techniques with and without the irrigation development project

Option name	Cropping patterns	Improved farming practices
No irrigation development project (donothing)	SR + SRR (in part SR + LR)	
With irrigation development project		
RiceRice	SR + LR	
RiceUpland	SR + SRR + LRU	
RiceRice+	SR + LR	WSRC + IRaP + WRS + mechanization
RiceUpland+	SR + SRR + LRU	WSRC + IRaP + WRS + mechanization

SR: Paddy rice cultivation in the short rainy season;
SRR: Ratoon rice cultivation after the short rainy season;
LR: Paddy rice cultivation in the long rainy season;
LRU: Cultivation of paddy rice and upland crops and in the long rainy season;
WSRC: Water Saving Rice Culture;
IRaP: Improved Ratoon Production;
WRS: Warehouse Receipts System.

Figure 1. Map of the Mwea Irrigation Scheme (adapted from JICA 2017)

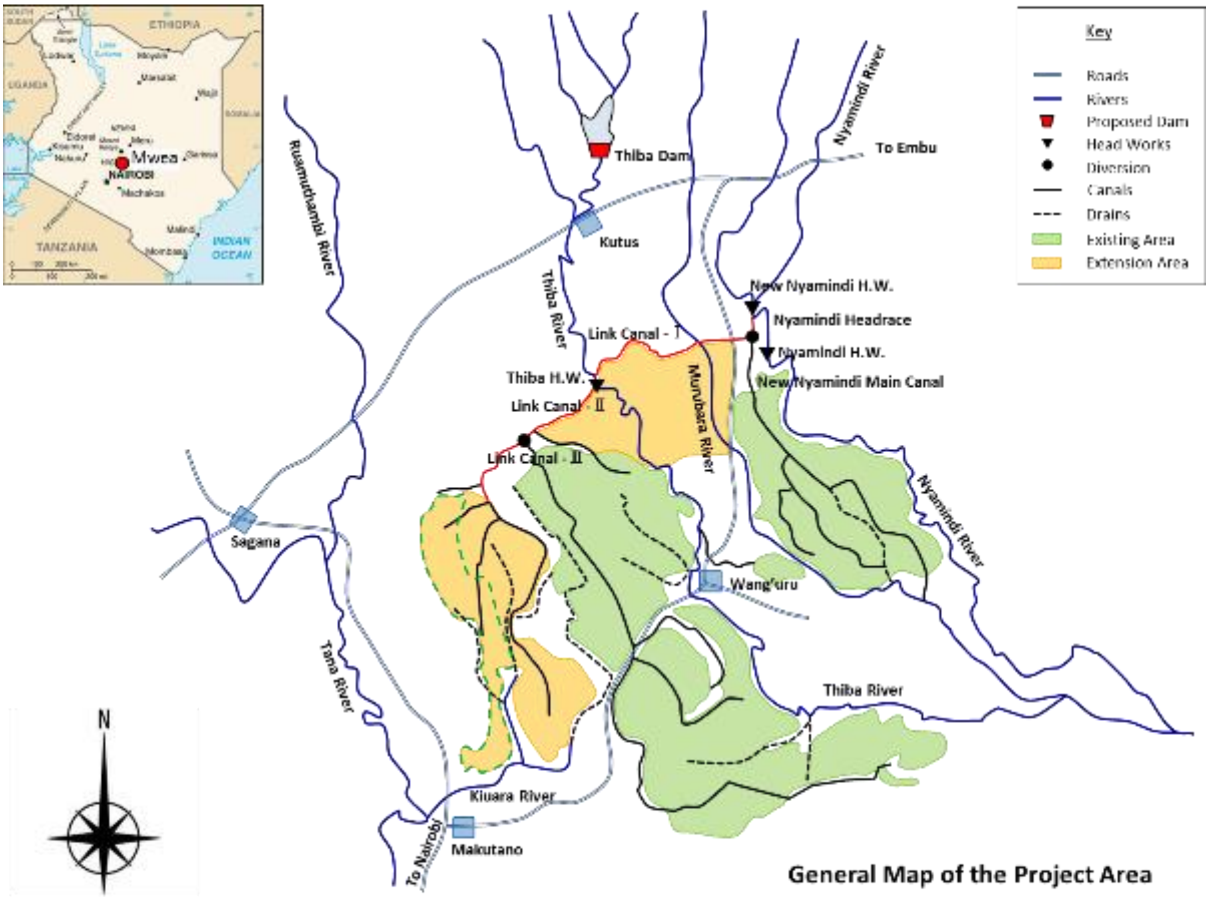

Figure 2. Evaluation of the project's effectiveness on climate change adaptation under uncertainty

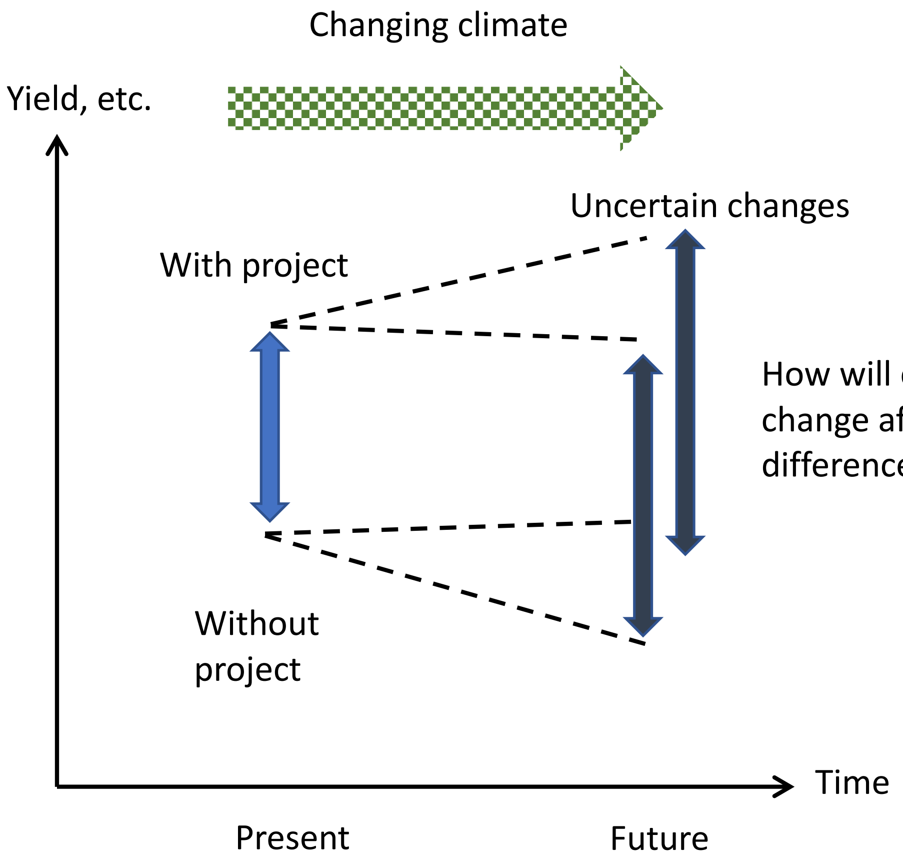

Figure 3. Flow of the simulation analysis

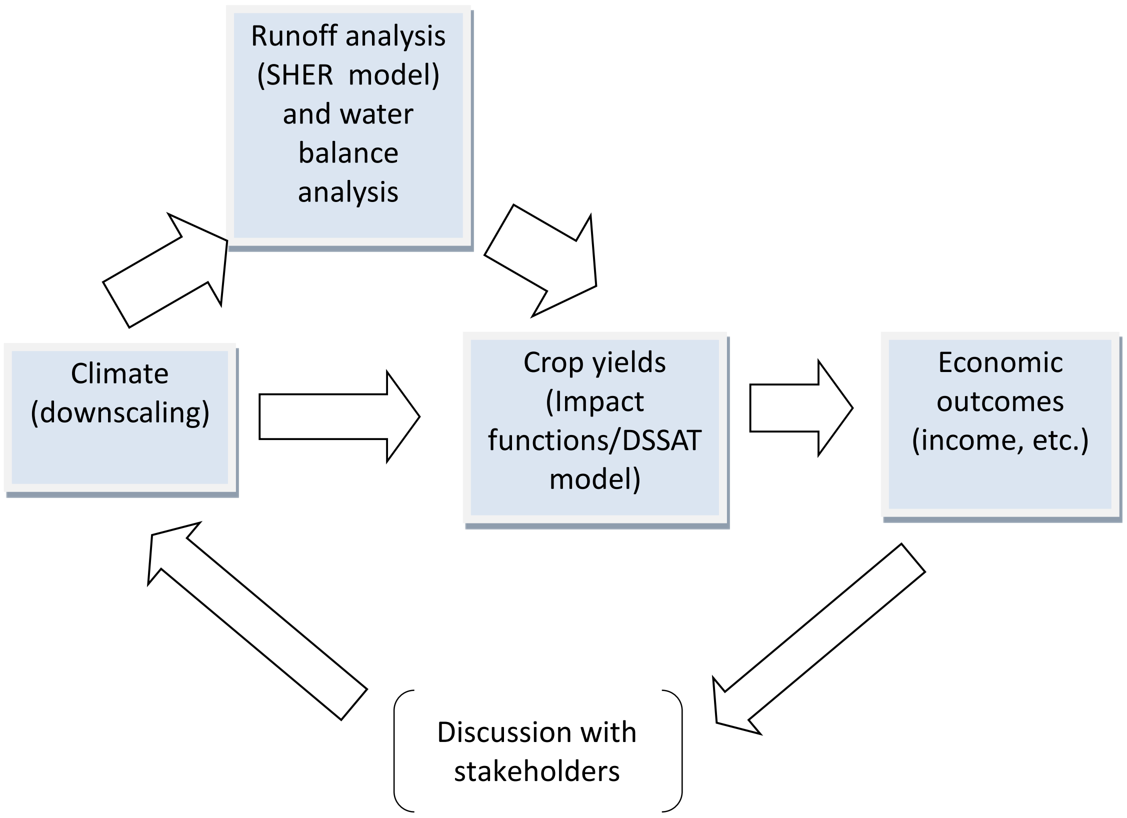

Figure 4. Future changes in annual temperature and precipitation in Mwea estimated by the delta change method using 14 GCM data (represented in ranges)

(a) Change of annual temperature relative to the baseline (1991-2010 average)

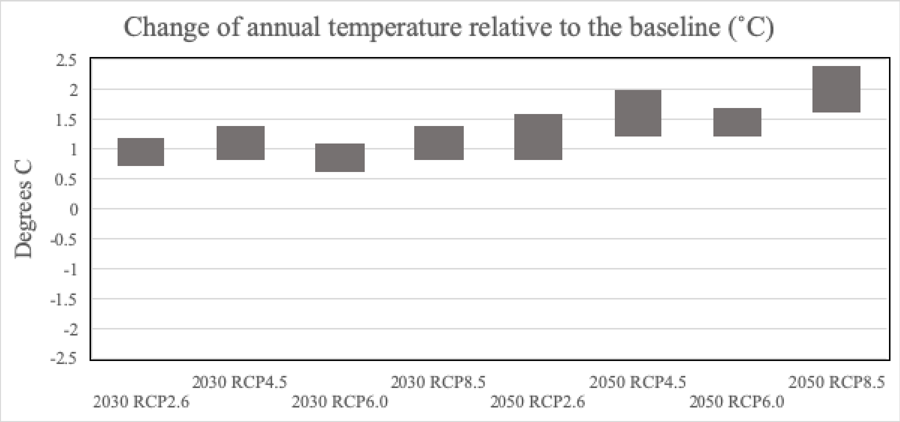

(b) Change of annual precipitation relative to the baseline (1991-2010 average)

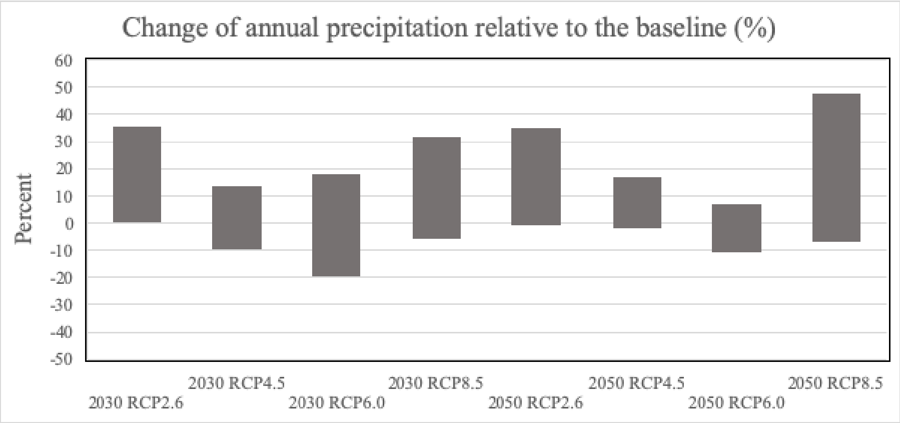

Note: See text for a description of the basis of the estimations.

Figure 5. Estimated mean river flows at the New Nyamindi Headwork for 2030 (left graphs) and 2050 (right graphs) for four seasons (Mar-Apr-May, Oct-Nov-Dec, Jan-Feb, and Jun-Jul-Aug)

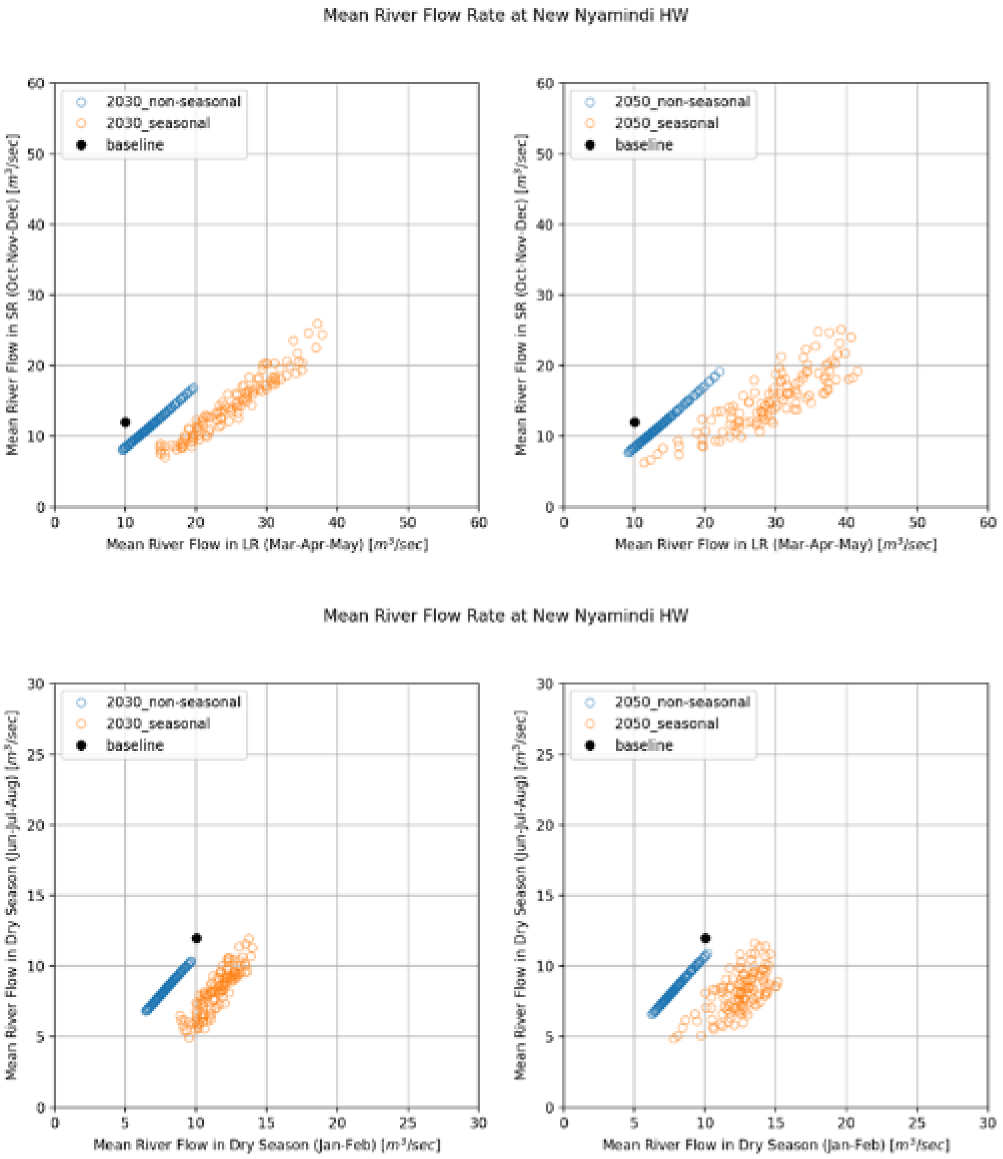

Note: The black dots show the baseline levels, and the blue and yellow circles represent the estimates without and with seasonality adjustments by approximation using a harmonic function (see Section 4.1. for our adjustment approach).

Figure 6. Simulation results of farmers’ annual income and the total rice production of the Mwea Irrigation Scheme (MIS)

(a) Farmers’ annual income

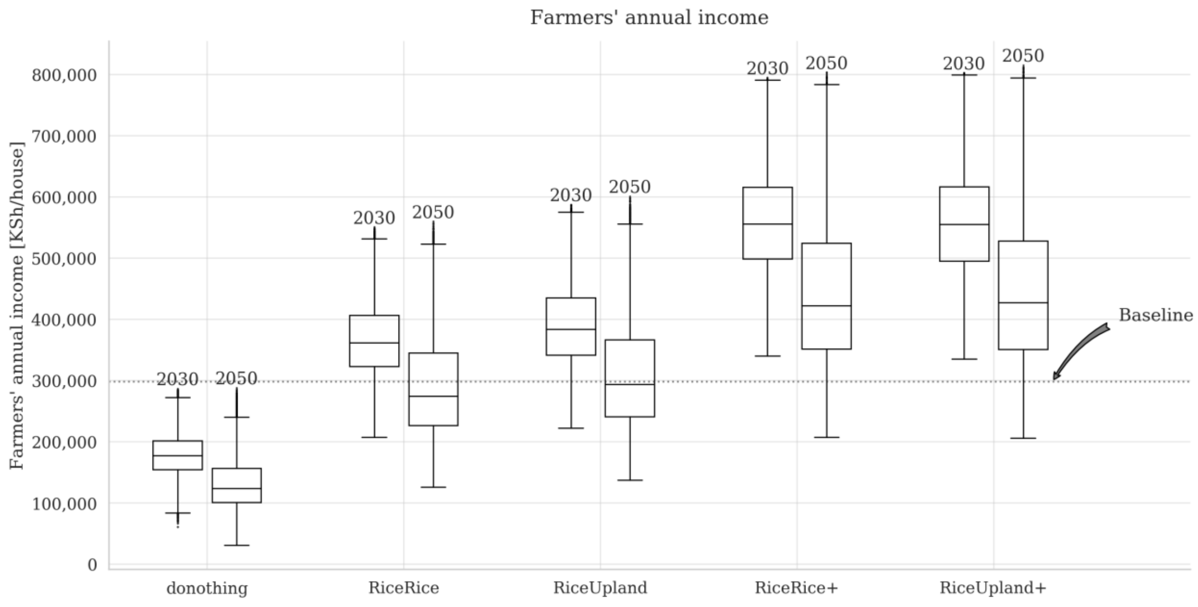

(b) Total rice production of the Mwea Irrigation Scheme (MIS)

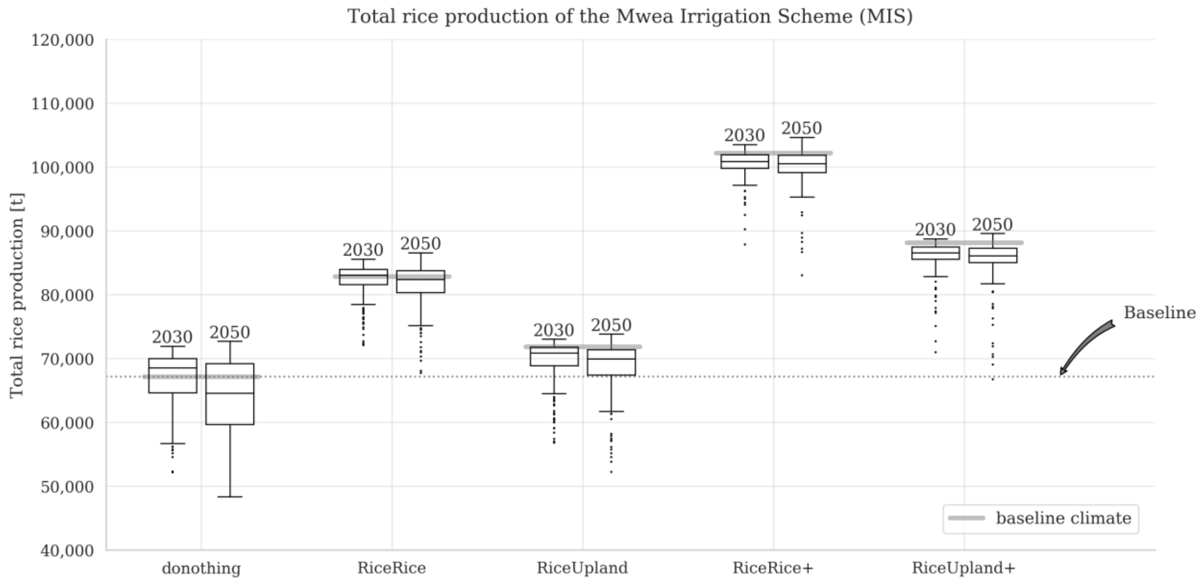

Note: The ends and middle lines of the boxes correspond to the quantiles (i.e., the middle line represents the median), and the whiskers represent the 1.5 interquartile ranges (IQRs). The dots above and below the whiskers represent the outliers.

Figure 7. Impact of climate change on the rice yield and average household income in the Mwea Irrigation Scheme (MIS) (percentage changes under climate change relative to the levels without climate change)

(a) Rice yield

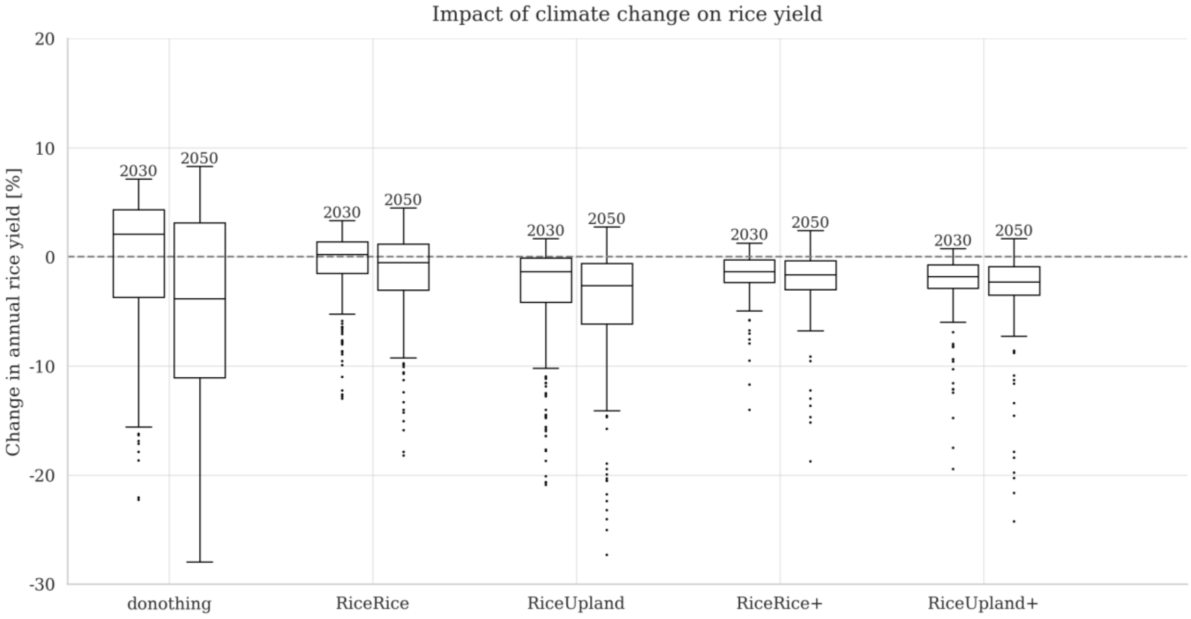

(b) Average household income

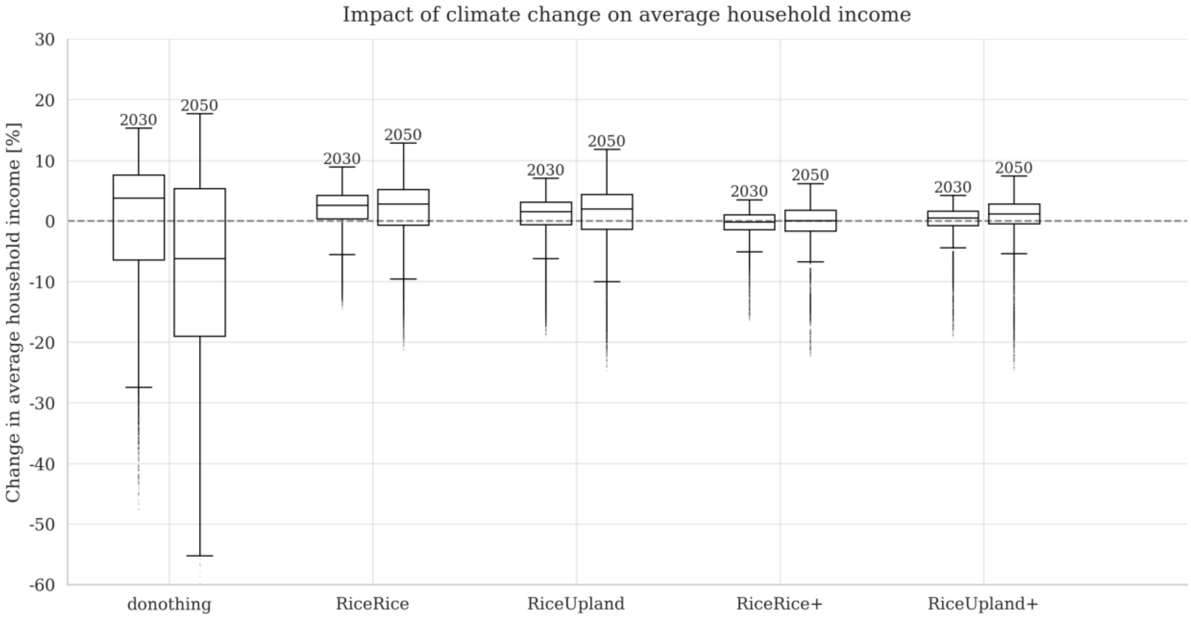

Note: The ends and middle lines of the boxes correspond to the quantiles (i.e., the middle line represents the median), and the whiskers represent the 1.5 interquartile ranges (IQRs).

Appendices

Appendix 1. Estimated crop coefficients

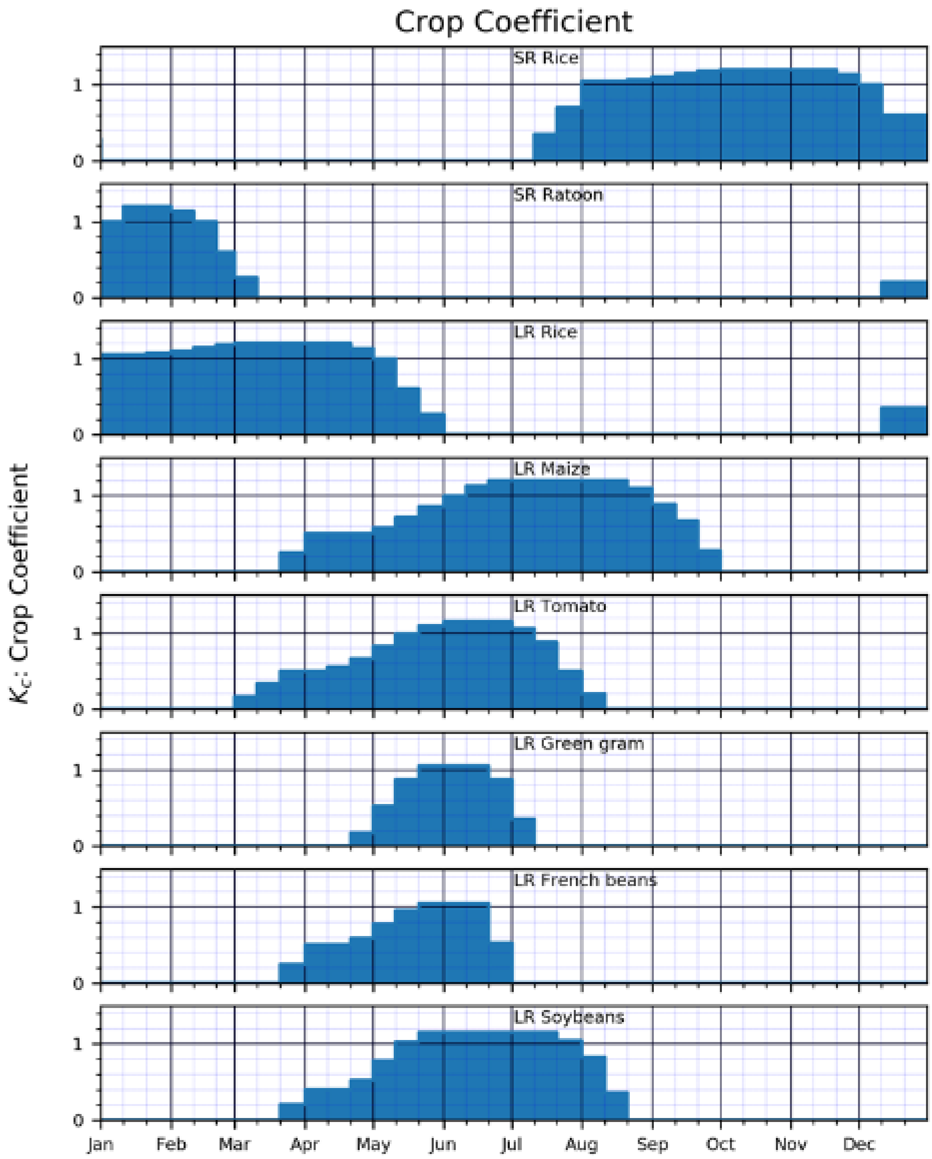

Appendix 2. Yield functions

Crop yields are first estimated by the DSSAT model in the conditions of Mwea for a set of combinations of temperature change and irrigation water sufficiency (7 x 11 = 77 combinations). By multivariate regression, the obtained values from the model are approximated in the following polynomial function of the percentage yield change from the baseline (i.e., the baseline/current yield with the respective water sufficiency rate), where *x* and *y* are the change in annual temperature from the present (˚C) and the irrigation water sufficiency rate (%), respectively:

$$f(x,y) = a_1 + a_2x + a_3y + a_4x^2 + a_5x^2y + a_6x^2y^2 + a_7y^2 + a_8xy^2 + a_9xy + a_{10}x^3 + a_{11}y^3$$

A table of coefficients by crop is presented below. Note that coefficients are different for different RCP scenarios because the estimates reflect the carbon dioxide fertilization effect.

SR: Short Rainy Season; LR: Long Rainy Season

Target Year	RCP	Crop	Parameter Estimates										
			a_1	a_2	a_3	a_4	a_5	a_6	a_7	a_8	a_9	a_{10}	a_{11}
2030	rcp26	SR Rice	1.16E+01	-3.14E+01	-2.97E-01	1.75E+00	-5.97E-02	2.29E-04	4.88E-03	-1.01E-03	3.77E-01	4.17E-01	-2.74E-05
		LR Rice	5.22E+00	-3.07E+01	1.23E-01	5.09E+00	-5.03E-02	2.29E-04	-2.21E-03	-6.43E-04	2.78E-01	-4.13E-01	8.19E-06
		LR French beans	1.04E+01	-1.22E+00	-3.50E-02	-3.78E-01	4.47E-02	-4.28E-04	9.19E-04	1.32E-03	-1.28E-01	-2.40E-01	-9.36E-06
		LR Maize	3.04E+00	7.76E+00	-2.67E-01	1.58E+00	3.56E-02	-3.36E-04	7.43E-03	2.94E-03	-4.01E-01	-6.17E-01	-5.10E-05
		LR Soybeans	1.14E+01	1.00E+00	-8.45E-02	-3.30E-01	4.97E-03	9.97E-06	2.69E-03	4.10E-04	-5.16E-02	7.25E-02	-1.91E-05
		LR Tomato	1.11E+01	-8.02E+00	-1.83E-01	-4.01E-02	-1.43E-02	1.55E-04	1.15E-03	-1.14E-03	1.38E-01	-1.71E-01	3.15E-06
	rcp45	SR Rice	1.34E+01	-3.24E+01	-3.06E-01	2.04E+00	-5.96E-02	2.07E-04	4.36E-03	-1.05E-03	3.89E-01	3.79E-01	-2.23E-05
		LR Rice	5.63E+00	-3.00E+01	9.22E-02	4.45E+00	-5.38E-02	2.70E-04	-1.46E-03	-8.01E-04	2.91E-01	-2.71E-01	3.66E-06
		LR French beans	1.14E+01	-8.12E-01	-7.51E-03	-6.83E-01	5.26E-02	-5.03E-04	4.19E-04	1.60E-03	-1.56E-01	-1.83E-01	-7.38E-06
		LR Maize	3.38E+00	7.46E+00	-2.90E-01	1.77E+00	2.95E-02	-2.78E-04	7.65E-03	2.71E-03	-3.77E-01	-6.52E-01	-5.12E-05
		LR Soybeans	1.24E+01	1.30E+00	-7.50E-02	-4.74E-01	8.99E-03	-1.71E-05	2.51E-03	4.17E-04	-5.52E-02	7.37E-02	-1.80E-05
		LR Tomato	1.21E+01	-7.75E+00	-1.82E-01	-2.52E-01	-1.12E-02	1.32E-04	1.08E-03	-1.07E-03	1.30E-01	-1.41E-01	3.52E-06
	rcp60	SR Rice	1.19E+01	-3.16E+01	-3.11E-01	1.64E+00	-6.83E-02	3.13E-04	5.06E-03	-1.28E-03	4.06E-01	4.50E-01	-2.80E-05
		LR Rice	6.50E+00	-3.13E+01	1.23E-01	4.89E+00	-6.00E-02	3.08E-04	-2.87E-03	-9.23E-04	3.17E-01	-3.26E-01	1.33E-05
		LR French beans	1.07E+01	-1.41E+00	-5.44E-02	-4.08E-01	4.15E-02	-4.12E-04	1.53E-03	1.30E-03	-1.21E-01	-2.08E-01	-1.39E-05
		LR Maize	3.34E+00	7.30E+00	-2.95E-01	1.90E+00	3.51E-02	-3.30E-04	7.74E-03	2.80E-03	-3.87E-01	-6.89E-01	-5.14E-05
		LR Soybeans	1.13E+01	1.44E+00	-6.95E-02	-5.86E-01	1.08E-02	-3.48E-05	2.33E-03	5.12E-04	-6.52E-02	9.78E-02	-1.67E-05
		LR Tomato	1.06E+01	-6.23E+00	-1.49E-01	-1.32E+00	6.70E-03	-4.24E-05	5.62E-04	-6.96E-04	8.94E-02	7.67E-02	5.96E-06
	rcp85	SR Rice	1.48E+01	-3.47E+01	-3.08E-01	3.33E+00	-7.36E-02	2.69E-04	4.84E-03	-1.03E-03	4.07E-01	2.53E-01	-2.75E-05
		LR Rice	7.36E+00	-2.99E+01	1.20E-01	3.90E+00	-5.59E-02	2.71E-04	-2.24E-03	-7.55E-04	2.96E-01	-1.08E-01	7.79E-06
		LR French beans	1.40E+01	-1.33E+00	-4.98E-02	-3.38E-01	5.10E-02	-4.98E-04	1.20E-03	1.55E-03	-1.47E-01	-2.46E-01	-1.21E-05
		LR Maize	3.86E+00	7.43E+00	-3.04E-01	1.82E+00	2.97E-02	-2.89E-04	7.95E-03	2.76E-03	-3.79E-01	-6.61E-01	-5.32E-05
		LR Soybeans	1.53E+01	1.01E+00	-8.36E-02	-4.01E-01	2.24E-03	3.83E-05	2.59E-03	2.45E-04	-3.26E-02	7.84E-02	-1.83E-05
		LR Tomato	1.37E+01	-6.22E+00	-1.84E-01	-1.47E+00	6.21E-03	-6.70E-05	1.11E-03	-5.54E-04	8.19E-02	1.56E-01	2.56E-06
2050	rcp26	SR Rice	1.27E+01	-3.41E+01	-2.92E-01	3.10E+00	-7.60E-02	3.30E-04	4.92E-03	-1.28E-03	4.21E-01	2.59E-01	-2.84E-05
		LR Rice	6.43E+00	-2.95E+01	5.10E-02	4.30E+00	-4.12E-02	1.48E-04	-4.26E-04	-5.32E-04	2.64E-01	-2.86E-01	-3.15E-06
		LR French beans	1.21E+01	-9.69E-01	-3.71E-02	-3.30E-01	4.96E-02	-4.43E-04	8.44E-04	1.38E-03	-1.44E-01	-2.83E-01	-8.59E-06
		LR Maize	3.50E+00	7.28E+00	-2.94E-01	2.08E+00	3.68E-02	-3.51E-04	7.94E-03	2.95E-03	-4.03E-01	-7.30E-01	-5.36E-05
		LR Soybeans	1.39E+01	8.90E-01	-9.08E-02	-3.55E-01	3.76E-03	2.85E-05	2.69E-03	2.75E-04	-3.80E-02	7.00E-02	-1.87E-05
		LR Tomato	1.25E+01	-6.76E+00	-1.80E-01	-7.71E-01	-9.42E-03	1.26E-04	1.01E-03	-9.21E-04	1.10E-01	-5.90E-02	3.99E-06
	rcp45	SR Rice	2.33E+01	-3.97E+01	-4.61E-01	4.02E+00	-1.32E-01	7.48E-04	6.23E-03	-2.69E-03	6.21E-01	3.88E-01	-3.15E-05

	LR Rice	1.36E+01	-3.08E+01	-2.17E-02	3.72E+00	-5.40E-02	2.50E-04	2.41E-04	-6.90E-04	3.04E-01	-7.93E-02	-7.13E-06
	LR French beans	1.90E+01	-1.41E+00	-2.55E-01	-6.99E-01	1.18E-02	-1.07E-04	2.76E-03	-2.70E-04	4.02E-02	-1.31E-01	-9.38E-06
	LR Maize	3.97E+00	8.03E+00	-3.06E-01	2.12E+00	3.18E-02	-2.87E-04	8.42E-03	2.83E-03	-3.98E-01	-7.85E-01	-5.72E-05
	LR Soybeans	2.08E+01	5.30E+00	-4.02E-01	-4.24E+00	-6.09E-03	1.52E-04	8.29E-03	-5.52E-04	3.59E-02	9.46E-01	-4.37E-05
	LR Tomato	1.84E+01	-6.21E+00	-2.10E-01	-1.14E+00	4.36E-02	-3.78E-04	8.74E-04	2.04E-04	-1.08E-02	-1.33E-02	6.66E-06
rcp60	SR Rice	1.96E+01	-3.61E+01	-4.28E-01	2.93E+00	-1.03E-01	5.42E-04	6.72E-03	-1.99E-03	5.15E-01	4.51E-01	-3.64E-05
	LR Rice	1.25E+01	-3.10E+01	4.97E-03	3.98E+00	-5.25E-02	2.29E-04	-1.57E-04	-6.19E-04	2.96E-01	-1.32E-01	-5.09E-06
	LR French beans	1.88E+01	-1.64E+00	-2.68E-01	-1.45E+00	9.48E-03	-1.28E-04	2.95E-03	-1.91E-04	5.04E-02	1.21E-01	-1.09E-05
	LR Maize	3.77E+00	8.01E+00	-3.04E-01	2.10E+00	3.11E-02	-2.76E-04	8.41E-03	2.80E-03	-3.96E-01	-7.81E-01	-5.72E-05
	LR Soybeans	2.05E+01	1.85E+00	-1.72E-01	-1.59E+00	5.27E-02	-5.22E-04	4.82E-03	1.93E-03	-1.81E-01	3.93E-01	-3.35E-05
	LR Tomato	1.78E+01	-6.89E+00	-2.28E-01	-7.27E-01	1.63E-02	-1.30E-04	1.36E-03	-4.94E-04	6.67E-02	-7.23E-02	3.49E-06
rcp85	SR Rice	3.19E+01	-3.77E+01	-6.07E-01	2.11E+00	-1.15E-01	7.29E-04	8.26E-03	-2.80E-03	6.10E-01	5.28E-01	-4.16E-05
	LR Rice	2.54E+01	-3.67E+01	-1.73E-01	4.86E+00	-1.06E-01	6.55E-04	7.34E-04	-2.13E-03	5.08E-01	-9.12E-02	-4.86E-06
	LR French beans	2.59E+01	-7.20E-01	-2.37E-01	-1.84E+00	2.21E-02	-2.54E-04	2.12E-03	3.52E-04	-2.95E-03	1.84E-01	-7.82E-06
	LR Maize	5.99E+00	7.40E+00	-3.90E-01	2.65E+00	2.82E-02	-2.54E-04	9.91E-03	2.67E-03	-3.84E-01	-9.00E-01	-6.48E-05
	LR Soybeans	2.87E+01	3.77E+00	-4.99E-01	-1.74E+00	2.30E-02	-8.48E-05	9.80E-03	-3.94E-04	3.49E-03	2.45E-01	-4.79E-05
	LR Tomato	2.58E+01	-7.45E+00	-2.68E-01	-6.83E-01	1.72E-02	-1.33E-04	7.83E-04	-5.69E-04	7.70E-02	-8.23E-02	9.95E-06

Appendix 3. Estimated irrigation efficiency levels in the MIS (adapted from JICA and Nippon Koei 2018)

(a) Without the irrigation development project

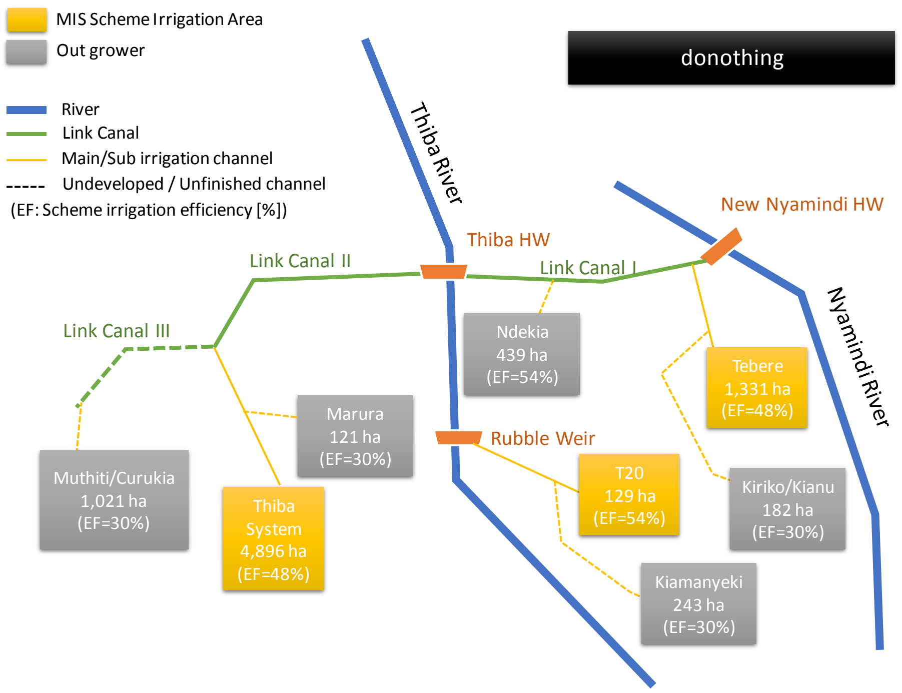

(b) With the irrigation development project

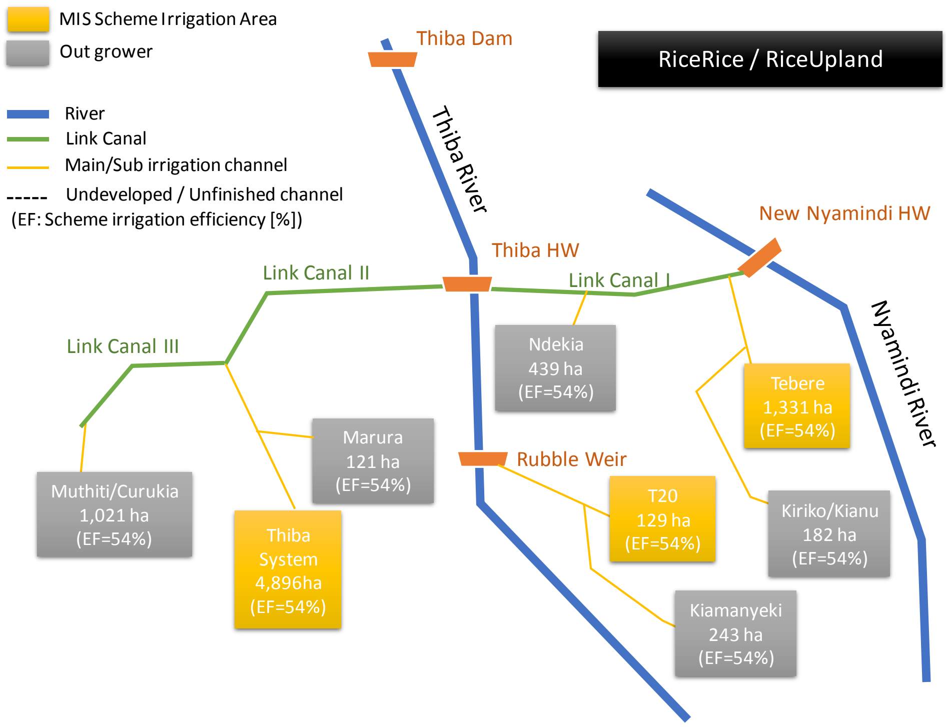

Appendix 4. Estimated wholesale prices of crops

Commodity	Unit Price (Ksh/kg)	
	Baseline*	2030**, 2050***
Rice (Basmati, short rain)	45	Upper bound: no change<br>Lower bound: 15% decrease
Rice (Basmati, short rain ratoon)	33	
Rice (Basmati, long rain)	60	
Dry maize	41	Upper bound: 10% increase<br>Lower bound: 10% decrease
Green gram	103	
Tomato	78	
Soybean	60	
French bean	31	

Note: * According to the Rice Mapp 2016 survey (Basmati), the Ministry of Agriculture, Livestock and Fisheries (dry maize, green gram, tomatoes), the JICA internal study (Soybeans, French beans)
** Growth rates set to be the same as those of the October 2017 World Bank Commodities Price Forecast.
*** Set to be the same as the 2030 levels.

Appendix 5. Impacts of climate change on the net present value of the irrigation development project for the four project options

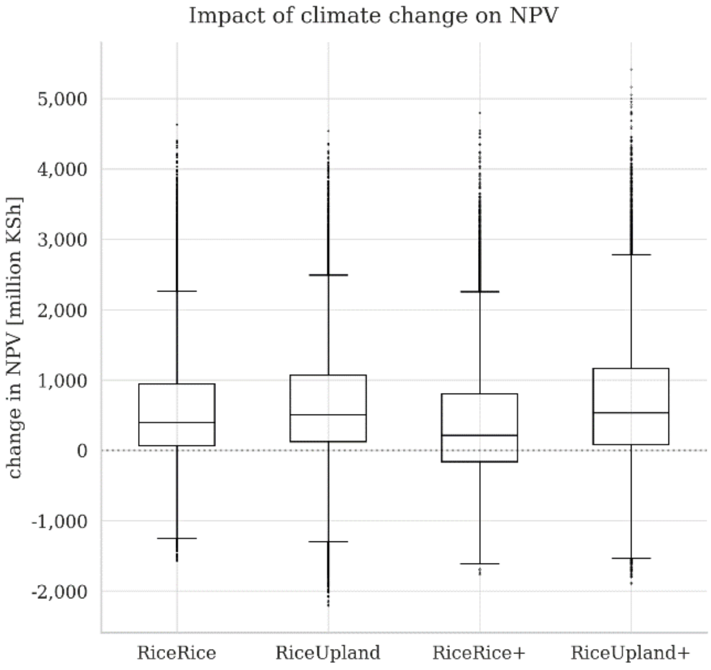

Note: Positive values mean that the project benefits are magnified by the inclusion of climate change damage in evaluation (i.e., representing the project benefits of climate change adaptation). The project lifetime is assumed to be 50 years.
