# Supplementary material for: Integrating economic measures of adaptation effectiveness into climate change interventions: A case study of irrigation development in Mwea, Kenya
Source: PLoS One. 2020 Dec 11;15(12):e0243779. doi: 10.1371/journal.pone.0243779 (PMC7732349; doi:10.1371/journal.pone.0243779)
Supplement: Attachment — Submitted filename: Response to Reviewers 1.docx [file pone.0243779.s006.docx]

Dear Editor,

We are very grateful for your review of our previous manuscript and would like to submit a revised version, now with a modified title “Integrating Economic Measures of Adaptation Effectiveness into Climate Change Interventions: A Case Study of Irrigation Development in Mwea, Kenya,” which reflects one of the reviewers’ comment.

We revised our manuscript by addressing the review comments. As they mainly concern the clarification of the study objective and the structure of the paper, our revisions are mostly focused on these aspects. Our responses to the reviewers’ specific comments are given below, in which the corresponding texts in the marked-up version are indicated.

Here we would also like to provide a note on how we intended to address the editorial issues highlighted in your last decision letter. First, to avoid the copyright problem of our previous Figure 1 (map), we have replaced it with our original map in the new version. Also, the roles of funders and employers (one of which is a private company) have now clearly been indicated in the relevant disclosure statements. Finally, about data accessibility, we are ready to make the data files open, for which we prefer to utilize the Supplementary Information page of the journal website. We are not sure how much size of data the journal website can accommodate (the size of our data is approximately 500MB), but we will follow your guidance when the paper becomes open as an article.

Thank you very much for your consideration.

Yours sincerely,

Daiju Narita, Ichiro Sato, Daikichi Ogawada and Akiko Matsumura

Reviewer #1: 

We are grateful for your comments on our last manuscript. We revised the paper based on the comments. Our responses (italic) to your specific comments (regular font) are as follows.

1-1. Although included in the abstract and at various points in the paper, “climate finance” is not clearly defined.

*We have inserted an additional statement explaining climate finance (Lines 48-51 of the marked-up version of the new manuscript). Climate finance is a commonly used term in finance without a unique and technical definition.*

1-2. The paper’s contribution could be more clearly explained within the introduction:
• First, I find your clear descriptions of models (climatic, hydrological, agricultural, and economic) to be a strong point of the paper. It seems others might want to replicate this methodological approach. Might you choose to explicitly present this feature as a selling point of your paper somewhere in the introduction section, perhaps in the paragraph beginning “Our study aims…” (p3)?

*Thank you for your comment. We have now revised the paragraph by following your suggestion (please see the third paragraph of the Introduction in the marked-up version of the new manuscript).*

1-3. • Secondly, the sentence beginning “However, to the authors’ knowledge…” (p3) is very complex, such that it makes it difficult to discern the paper’s unique contribution. Is it the economic appraisal of irrigation as a climate change adaptation strategy? I believe others have done that already. (For example, Cunha et al. 2015 model agricultural incomes in Brazil, and Finger et al. 2010 model economic outcomes in Switzerland.) If the paper’s contribution is that it is the first to model the economic aspect *of a specific irrigation project* as a climate change adaptation strategy, this could be clarified with a more direct/simplified sentence. I’d also like a bit more information about how your paper differs from those process-based and statistical models you mention.

*Following your comment, we substantially revised the corresponding fourth paragraph of the Introduction (please see the marked-up version of the new manuscript). Your suggested papers are now also cited.*

1-4. Based on the introduction section, I expected the paper to discuss economic outcomes on a project-level scale; instead, the economic results were presented at the scale of household income. If I’ve correctly understood that project-level economic outcomes are one of the paper’s main contributions to the literature, I suggest creating some version of Appendix 5 as a main figure within the paper and discussing it in detail. Appendix 5 isn’t quite right for this purpose, though, as it appears to subtract the NPV that doesn’t account for climate change from the NPVs that account for climate change. Have you presented the NPVs under climate change anywhere? Phrasing like “project appraisal of public investments” (p15) led me to expect this as a principal finding.

*In response to your comment, we have now inserted a new figure (Figure 8) that shows the NPV for different project options, with and without the effects of climate change. The results are consistent with (have no marked differences from) the other results we show (Figures 6 and 7).*

1-5. I do not understand the “preliminary” and “main” stages of analysis described on p6 and in Figure 3. Did you only run the models for cropping strategies of interest? Clarification would be useful.

*We added text (Lines 223-225 of the marked-up version of the new manuscript) to explain more about the purpose of the preliminary analysis.*

1-6. How did you select the four RCP scenarios (p7)? How many RCP scenarios exist?

*There are four RCP scenarios, and our analysis deals with all of them. In the revised text, we rephrased the text (Lines 280-282 of the marked-up version of the new manuscript) and made it more explicit (“which are comprised of…”).*

1-7. For the economic analysis, could you justify your apparent assumption that the UN’s projected % increases in the rural population will be equal to the % increase in the farming population (and, therefore, the amount of land available to each farming household)?

*We drew on the expected growth rate of Kenya’s national population because there are no population projections specific to Mwea. On this point, we revised the corresponding text (Lines 515-516 of the marked-up version of the new manuscript).*

1-8. I suggest improving the presentation of results as follows:
• The project area map (Figure 1) would benefit from a scale.

*The map was updated and is now shown with a scale.*

1-9. • In Figures 4a and 4b, it would be useful to darken the baseline. It would also be useful to make it clearer that the results are not temporal by clearly differentiating the 2030 values from the 2050 values. My first inclination was to read the bars from left to right, as if they progressed over time.

*We updated the figure by adopting your comment.*

1-10. • For Figure 5, it is not clearly explained that the rainy seasons are together in one panel, and the dry seasons are together in the other panel. Might you briefly explain the benefit of grouping the seasons in this way? It would also be useful to write out “long rains” and “short rains” on the axis labels.

*Following your comment and to enhance readability, we updated the figure and figure caption. We grouped graphs purely for the sake of compact representation, and with additional labels and caption text, we believe that the presented information has become more understandable to the readers than the previous version’s.*

1-11. • For Figure 6, it is not clearly specified what the baseline represents. Current annual income and rice production? With data from which year?

*We have now inserted a note explaining what the baseline represents (Lines 585-589 of the marked-up version of the new manuscript as well as in the figure caption).*

1-12. Smaller points of clarification:
In the first introductory paragraph, it is unclear whether the UNEP-DTU estimates are the costs necessary to fully mitigate losses or are actual predicted expenditures in developing countries.

*The Adaptation Finance Gap Report (UNEP-DTU 2016) estimates adaptation costs as the costs of achieving societally set adaptation targets or goals. Thus, they are not the costs necessary to fully mitigate losses but are actual predicted expenditures in developing countries to achieve their respective adaption goals, which reflect national circumstances including fiscal resource limitations. Thus the following revised sentence is suggested to make that clear (Lines 42-43 of the marked-up version of the new manuscript).*

*“UNEP-DTU (2016) estimates that annual costs in 36 developing countries to meet their adaptation targets will range from US$140 to 300 billion by 2030, ...”*

1-13. On the bottom of p5, the authors describe feeding simulation outputs of each model to another rather than building a single model. Are there downsides to this approach? Is this common practice (citations)?

*We have inserted an additional remark (Lines 198-202 of the marked-up version of the new manuscript) on the implications of our methodological choice of modeling. The approach itself is not particular and is not related to specific citations.*

1-14. On p6, when describing the RDM framework, it would be useful to briefly summarize the framework again rather than referring the reader back to the introduction section.

*We inserted a new footnote (Footnote 3) as a brief explanation of the RDM framework.*

1-15. Might something like “no irrigation” be a clearer scenario descriptor than “do nothing”?

*Some basic infrastructure of irrigation already exists in the study area (Mwea), and so we think that the name “no irrigation” could be misleading. So we would like to keep the original descriptor “do nothing.”*

Reviewer #2:

We are grateful for your comments on our last manuscript. We revised the paper based on the comments. Our responses (italic) to your individual comments (regular font) are as follows.

General comment

The manuscript deals with a very important subject involving climate change adaptation which is a contemporary area of research. Irrigation as climate change adaptation strategy is a fundamental subject and finding of the study. Though comprehensive, appropriate and relevant data have been collected and analysed, the authors need to improve a number of things in the manuscript including the title, explicit statement on objective of the study, clear separation of results and methodology so that comprehensive presentation of results and discussion are consolidated in one section. The methodology is not reported in past tense to indicate that it is a report of how the research was pursued to generate the results presented. Instead it is presented in present and continuous tenses as if it is not a report of how the research was pursued. The use of ""We" is also monotonous and is not encouraged. The conclusion need to be revisited and made more explicit in addressing the objective of the study. Generally the manuscript requires elaborate editing and synthesis. The details of the review are marked on the pdf format of the paper attached.

*Thank you for your comment. We revised our manuscript by mostly adopting your comments. Our responses to your specific points are given below. As for your general editorial comments such as about the tense and the replacement of the pronoun “we,” we would like to keep our editorial choices as they have been made through consultation with a native-speaker expert of academic English, and in fact, we’ve followed the common practices of some academic disciplines (such as economics).*

2-1. (Multiple comments on the article title)

*Reflecting your comments, we revised the title as follows: “Integrating Economic Measures of Adaptation Effectiveness into Climate Change Interventions: A Case Study of Irrigation Development in Mwea, Kenya”*

2-2. p.8. The abstract needs to be refined to include issues on methods as well as policy and contribution to knowledge

*Following your comment, we revised the abstract to better explain the scope of the paper, which is a demonstration of project-specific financial evaluation of climate change adaptation integrating multiple types of modeling. Please also see the marked-up version of the new manuscript.*

2-3. p.8 General comments: The study objective is not explicitly given. I am made to imagine that the authors wanted to advance the importance of climate financing, but this hasn't come out clearly. Is climate financing, nascent concept in climate change adaptation and the need for economic assessment of various adaptation strategies i.e. under irrigation including choice of enterprise and other intervening variables critical ?. if so you need to have framed your objective around the need for generic versus specific economic evaluation in choice of adaptation measures and adaptation financing. This will aptly capture the theme on effectiveness as intended

*Following your comment, we significantly updated the Introduction to better explain the objective of analysis, with some more reference to the climate finance concept. In short, the scope of the paper is a demonstration of project-specific financial evaluation of climate change adaptation integrating multiple types of modeling, for which the needs of methodology exist in the field of climate finance (i.e., finance on climate change mitigation and adaptation measures). Please also see the first three paragraphs of the Introduction in the marked-up version of the new manuscript.*

2-4. p.8. what are some of the drivers towards attention to '' efffectiveness''

*We elaborated the sentence to better describe the problem (Lines 54-57 of the marked-up version of the new manuscript).*

2-5. p.9. is it gaps or needs?

*It is “needs.” We elaborated the preceding discussion of methodological needs in the context of climate finance (the second paragraph of the Introduction in the marked-up version of the new manuscript).*

2-6. p.9. are you performing or providing a case study?

*To make it clear, we rephrased the text as “demonstrate a quantitative evaluation…” (Lines 76-77 of the marked-up version of the new manuscript).*

2-7. p.9. citation missing

*A citation has been inserted (Line 104 of the marked-up version of the new manuscript).*

2-8. p.9. project evaluation is so broad, so you need to simplify and state what aspect of project evaluation you are focusing on .i.e. financial, economic, social, etc.

*We reworded it as “project-specific evaluation.” Also, the revised introduction now stresses the implications of this study in the context of finance.*

2-9. p.10. Take to relevant ( results) section

*Thank you for your suggestion. But we would like to keep the structure here as in some academic disciplines (e.g., economics), whose readership we target, adopts the convention that a brief summary of results is given in the Introduction, just we did.*

2-10. p.10. delete

*Deleted in the new version.*

2-11. p.10. central Kenya?

*The word “central” is now inserted.*

2-12. p.10. source??

*A citation is now inserted.*

2-13. p.12. see comments in 3 above. It is advisable that you organize this section as follows

defining the problem

quantitative formulation of the various models

model verification and sensitivity analysis

model validation

*Thank you for your suggestion. For the new version, we did move numerical results from Section 4 to Section 5, but since the last two categories you suggest do not match with the structure of our analysis, we decided not to fully adopt your suggested organization. Still, we appreciate your comment and reflected it wherever we can -- for example, the section title “Evaluation Approach” is now changed to “Defining the Problem” by adopting your suggestion.*

2-14. p.13. define where i.e. http site

*The URL is now inserted.*

2-15. p.14. Are these not results that should appear in the results and not methodology section.

2-16. p.14. In this section methodological issues are mixed with results. For example what is presented from line 43 based on Figure 5 are results. You need to separate the results from the methodology.

*Thank you for the comments. Now these discussions are moved to the Results section.*

2-17. p.19. it's better to capture this in the methodology section

*Thank you for your comment, but we are in fact puzzled by this remark, as it seems inconsistent with what you suggest in the preceding comments. Since this part deals with results of model simulations, we would like to keep it in this section.*

2-18. p.20. what is the importance of this ? ( higher variance has policy implication which you need to comment on

*We added some more clarification on this result (Lines 601-602 of the marked-up version of the revised manuscript).*

2-19. p.20. what is the plausible explanation for this?

2-20. p.20. Is it positive income changes due to climate change and not climate change adaption through irrigation.

*To make it clear, we have revised the corresponding text as “positive income changes even under climate change, reflecting improvement and diversification of crop farming” (Lines 622-623 of the marked-up version of the revised manuscript).*

2-21. p.20. Do you mean to or of?

*The text of the previous version might have lacked clarity generally on this part, and we revised the corresponding text as “… -- this corresponds to the greater uncertainty of climate change in the former year…” (Line 628 of the marked-up version of the revised manuscript).*

2-22. p.20. Delete "a".

*The typo has been corrected.*

2-23. p.20. Please redo the conclusion to capture the study objectives and major findings and avoid repetitions

2-24. p.21. This needs rewording as it is a copy paste of the introductory section

2-25. p.21. This is repetition of the previous statements above

*Following your comments, we significantly updated the text throughout the conclusion part.*
