# Supplementary material for: Integrating economic measures of adaptation effectiveness into climate change interventions: A case study of irrigation development in Mwea, Kenya
Source: PLoS One. 2020 Dec 11;15(12):e0243779. doi: 10.1371/journal.pone.0243779 (PMC7732349; doi:10.1371/journal.pone.0243779)
Supplement: Attachment — Submitted filename: Response_letter_Nov2020 2_.docx [file pone.0243779.s007.docx]

Dear Editor,

We are grateful for your additional review on the last version of the manuscript. We revised it by following your comments and would like to submit a new version. Our responses to your specific comments are as below (please note that in a marked-up version of our resubmitted manuscript, our changes corresponding your comment 3 are not highlighted in red, as the changes are essentially only on formatting and are not easy to be displayed well in a marked-up mode of MS Word).

Thank you very much for your consideration.

Sincerely,

Daiju Narita, Ichiro Sato, Daikichi Ogawada and Akiko Matsumura

<Responses to your specific comments>

*1. Authors should break the paragraph in Section 4.2 to at least 2 or 3 to enhance readability. Other paragraphs in the introduction and other sections are also too long and should be cut to 2 or more paragraphs.*

We broke the paragraphs of Section 3.2 (formerly Section 4.2) and the others long paragraphs (the introduction and other sections) in the new version to enhance readability.

*2. Authors have to remove the section on page 4 from "Our analysis reveals that climate change reduces ....to The simulation results are shown in Section 4. while Section 6 discusses them and concludes the paper. The first paragraph is not required to pre-empty the findings as this is reserved for the Results section and outlining the sections is not at all necessary.*

Theses paragraphs were removed in the new version.

*3.Authors should stick to the PLoS referencing system or at least other published papers to understand the proper referenceing for the jounral. For example, where consecutive papers are cited authors are using for e.g (10), (11), (12), (5), (13), (14) instead of the standard (5, 11-14) or even (14-15). This should be corrected throughout the paper. There is also overuse of the word e.g when referencing papers which is not appropriate way of citing papers and should be removed or used to the minimum when necessary.*

The numbering problem of references occurred due to our unfamiliarity with this referencing system. We are sorry about this and have corrected the problem for the new manuscript. Also, the word “e.g.” was not indispensable in most parts, and these were deleted in the new version.

*4. The study area and the Mwea Irrigation Development Project should come as a sub-section under Methods section. It is also not standard to have so many lines as one paragraph, break to various paragraphs.*

In the new version, we moved this part to the Methods section and made it a subsection. We also broke its paragraphs to enhance readability.

*5.There is no conclusion and authors should separate the Discussion and Conclusion provide a proper conclusion highlighting key messages and learning points from the paper. Few recommendations for building resislience will also be good for the conclusion.*

In the new version, we separated a Discussion section and a Conclusion section and included some new text in the latter on key messages and learning points. We also inserted a remark that our study offers some general hint that irrigation may enhance the local capacity to deal with the worst possible outcomes of climate change.
